# Effect of dietary calcium and vitamin D supplements on plasma bone turnover biomarkers, bone mineralization, bone strength, and lameness score in gilts

**DOI:** 10.1093/jas/skae310

**Published:** 2024-10-15

**Authors:** Thomas S Bruun, Søren K Jensen, Torben Larsen, Mai Britt F Nielsen, Laurent Roger, Takele Feyera

**Affiliations:** SEGES Innovation, Aarhus, Denmark; Department of Animal and Veterinary Sciences, Aarhus University, AU-Viborg, Tjele, Denmark; Department of Animal and Veterinary Sciences, Aarhus University, AU-Viborg, Tjele, Denmark; SEGES Innovation, Copenhagen, Denmark; dsm-firmenich Animal Nutrition and Health, Neuilly-Sure-Seine, France; Department of Animal and Veterinary Sciences, Aarhus University, AU-Viborg, Tjele, Denmark

**Keywords:** bone mineralization, bone strength, calcium, growing gilt, lameness, osteocalcin

## Abstract

This study investigated the impact of calcium (**Ca**) and vitamin D supplements on bone metabolism, bone measurement, lameness, and selection rate in gilts fed 5 dietary treatments. Two Ca levels (6.85/6.42 [adequate; **ACa**] or 8.99/8.56 [high; **HCa**] g/kg) were combined with either 856 IU/kg vitamin D3 (Danish feeding standards; adequate; **AD3**) or 50 μg/kg 25-hydroxyvitamin D3 (high; **HHyD**) to create **ACaAD3**, **HCaAD3**, **ACaHHyD**, and **HCaHHyD** diets. The values 6.85/6.42 and 8.99/8.56 g/kg correspond to adequate and high Ca supply for gilts weighing 32 to 100 and 100 to 180 kg body weight (**BW**), respectively. The fifth diet was a combination of HCa and 2,000 IU/kg vitamin D3 (high; **HD3**) to create **HCaHD3**. Two hundred gilts were phase fed the dietary treatments from 32 to 100 and 100 to 180 kg BW until they were slaughtered, either at 100 or 180 kg BW. The gilts were weighed fortnightly, and plasma and urine samples were collected at 100 and 180 kg BW. At slaughter, the 2^nd^ and 3^rd^ metacarpal bones were collected for bone parameters measurements. Lameness and selection rate were assessed within the last 7 d at 100 and 180 kg BW. Dietary treatments did not affect gilts’ growth performance and plasma concentration of Ca, but the urinary concentration of Ca was greater in HCa-supplemented gilts at both 100 (*P* = 0.003) and 180 (*P* = 0.05) kg BW. Plasma concentration of vitamin D3 (*P* < 0.001) and 25-hydroxyvitamin D3 (*P* < 0.001) showed dose-dependent responses at both 100 and 180 kg BW. Bone-specific alkaline phosphatase was greater (*P *= 0.02) in the plasma sample collected at 180 kg BW in gilts fed the HCaHD3 diet and tended to be greater in gilts fed the ACaAD3 diet (*P* = 0.06). The bone ash content (*P *= 0.02) was greater in gilts fed the HCaAD3 diet and slaughtered at 100 kg BW compared with gilts fed the ACaAD3 and ACaHHyD diets. However, bone weight, length, thickness, dry matter, and mineral content did not differ among the dietary treatments at both 100 and 180 kg BW (*P* > 0.05). Neither lameness nor selection rate was affected by the dietary treatments. The average daily gain of gilts weighing 32 to 100 and 100 to 180 kg BW showed a positive correlation with bone strength (*r *= 0.37; *P* < 0.001) and bone ash content (*r* = 0.24; *P* = 0.02), respectively. In conclusion, higher Ca and vitamin D3 supplementation slightly increased bone ash content but had no effect on the lameness or selection rate of the gilts compared to those fed according to the Danish nutrient standards.

## Teaser

The study indicated that increasing calcium and vitamin D3 levels above the Danish recommendations increased the concentration of bone-specific alkaline phosphatase in plasma, although it did not show any significant improvement or deterioration in the lameness score or selection rate of the gilts.

## Introduction

Lameness is of growing concern in breeding sows. This issue not only present significant economic, welfare, and health challenges but also frequently leads to the premature culling of sows (Willgert et al., 2014; Ala-Kurikka et al., 2017). In a survey across 17 Danish production herds, it was found that lameness accounts for 40% to 70% of culled sows in these herds and is a significant contributor to the involuntary culling of breeding sows (Vestergaard et al., 2016). Furthermore, the authors observed greater incidences of lameness in 1^st^ and 2^nd^ parity sows compared with older counterparts. Leg weakness during the growing phase exerts a detrimental impact on bone development and the overall longevity of breeding sows (Anil et al., 2009; Pluym et al., 2013). The structural integrity of the legs is closely associated with calcium (**Ca**) and phosphorus (**P**) reserves in the bones (Schlegel and Gutzwiller, 2020). Nutrition is crucial for bone development and maintenance, so inadequate mineral supply or concentrations can impair bone quality and increase the risk of lameness (van Riet et al., 2013). Intervening with dietary strategies during the growth phase is crucial in enhancing bone development and strength, and Ca plays a pivotal role in this regard (Szulc and Bauer, 2013). Efficient bone mineralization requires adequate Ca absorption from the gut and maintenance of optimal levels of serum Ca, which is tightly regulated by vitamin D in the body (EFSA, 2009; Goltzman, 2018). Lauridsen et al. (2010) observed a lower bone strength in gilts after feeding increasing levels of 25-hydroxyvitamin D (5, 20, 35, and 50 μg/kg) from the first estrus until day 28 of gestation at a constant level of Ca compared with equivalent levels of vitamin D3, and they observed the greatest bone strength at a dietary level of 800 IU/kg of vitamin D3. It is well known that Ca and P interact with each other, and a high Ca intake may reduce P availability (Stein et al., 2011), and potentially also interact with high vitamin D, which may ultimately impede bone mineralization and strength (Lauridsen et al., 2010). This may be further reinforced by diets that often contain more Ca than formulated due to an oversupply of limestone (Lagos et al., 2023). Moreover, a high level of dietary Ca can negatively affect the digestibility of P (Hu et al., 2022; Lee and Stein, 2022), and therefore, less P will be available for storage in bone and muscle. Furthermore, the use of 25-hydroxyvitamin D enhances Ca absorption (O’Doherty et al., 2010), potentially leading to an unfavorable shift in the ratio of absorbed Ca to P.

It was hypothesized that increasing Ca and vitamin D levels beyond the Danish feeding standards for growing gilts, at an adequate level of digestible P, may potentially diminish bone mineralization and strength due to impaired P utilization, consequently increasing the prevalence of lameness without affecting average daily gain (**ADG**) and feed conversion efficiency of the gilts. Hence, the study investigated the impact of varying Ca and vitamin D3 levels, and 25-hydroxyvitamin D3 at fixed levels of dietary P on plasma bone turnover biomarkers, vitamin D3 and its metabolites, bone mineralization and strength, lameness, and selection rate in gilts fed the dietary treatments from 32-180 kg body weight (**BW**).

## Materials and Methods

The procedures for blood sampling and the housing of animals complied with Danish laws and regulations for the humane care and use of animals in scientific research [The Danish Ministry of Justice, Animal Testing Act (Consolidation Act number 726 of September 9, 1993, as amended by Act number 1081 on December 20, 1995)]. The study protocol received approval (License no. 2021-15-0201-00971), and the Danish Animal Experimentation Inspectorate supervised the experiment.

### Animals, randomization, and housing

Two hundred DanBred hybrid gilts (Landrace × Yorkshire) were included in this experiment. The gilts were purchased from a multiplier herd with an average BW of 32.4 ± 5.9 kg and an approximate age of 80.3 ± 2.6 d. The gilts were descended from various litters including 50 sows and 30 boars. The randomization of gilts into dietary treatments was carried out in 3 steps. Initially, 5 gilts from 20 flocks of siblings were allocated to be slaughtered at 180 kg BW, allowing one gilt from each litter to be fed one of the dietary treatments. Secondly, the remaining 100 gilts to be slaughtered at 100 kg BW were randomized to the 5 treatment groups, taking the boar into consideration. Finally, to standardize BW within the treatment groups and to minimize BW variation at slaughter, gilts were allocated to 5 BW groups according to their initial BW and accordingly randomized to the dietary treatments. Therefore, in each group, 20 gilts were initially assigned to be slaughtered at 100 and 180 kg BW.

The gilts were initially housed in a group of 8 gilts per pen (1.9 × 4.3 m) on a partially slatted floor, and each pen was equipped with a water drinking cup and a feeding station (1.7 × 0.6 m). All gilts in a pen received the same dietary treatment. After the first slaughter BW, the number of gilts per pen was reduced to 4. Climate regulation within the housing facility was achieved through negative pressure via wall inlets, and the housing facility temperature was set at 20.0 °C. The light was turned on from 0700 to 1600 hours, and a small, dimmed light was installed in all feeding stations to guide the gilts toward the feeding station during the periods of darkness.

### Diet and feeding

Gilts were fed a barley-, wheat-, and soybean meal-based diet (32 to 100 kg BW) or a barley-, wheat-, and oat-based diet (100 to 180 kg BW) optimized to meet the Danish nutrient standards (Tybirk et al., 2021) for gilts weighing 32 to 100 and 100 to 180 kg BW (**Table 1**). Danish nutrient standards for gilts, developed using SID Lys and SID CP to regulate the ADG and feed utilization, incorporated a higher safety margin for Ca and P than nutrient standards for slaughter pigs to enhance bone ash content and strength (Tybirk et al., 2014). The rates of inclusion for wheat, soybean meal, and palm oil were adjusted slightly to maintain a constant net energy level among the 5 dietary treatments. Thus, all diets were formulated to be both isoenergetic, expressed as potential physiological energy that a closely aligned with the net energy system (Patience, 2012), and isonitrogenous.

**Table 1.** Dietary ingredients and calculated chemical compositions of the experimental diets used for gilts weighing 32 to 100 and 100 to 180 kg body weight

Item	32 to 100 kg body weight^1^					100 to 180 kg body weight^2^				
	ACaAD3	HCaAD3	ACaHHyD	HCaHHyD	HCaHD3	ACaAD3	HCaAD3	ACaHHyD	HCaHHyD	HCaHD3
*Dietary ingredient, %*										
Barley	50.0	50.0	50.0	50.0	50.0	50.0	50.0	50.0	50.0	50.0
Wheat	27.8	26.7	27.7	26.8	26.6	21.1	19.7	20.5	19.7	19.5
Oat	5.00	5.00	5.00	5.00	5.00	10.0	10.0	10.0	10.0	10.0
Sugar beet pellets	3.00	3.00	3.00	3.00	3.00	6.00	6.00	6.00	6.00	6.00
Soybean meal, dehulled	9.69	9.78	9.72	9.80	10.0	7.32	7.71	7.70	7.71	7.83
Palm oil	1.52	1.81	1.52	1.82	1.83	3.25	3.67	3.41	3.67	3.67
L-Lys	0.28	0.28	0.28	0.28	0.27	0.03	0.03	0.03	0.03	0.02
DL-Met	0.04	0.04	0.04	0.04	0.04					
L-Thr	0.08	0.08	0.08	0.08	0.08					
Monocalcium phosphate	0.67	0.67	0.67	0.67	0.67	0.35	0.35	0.35	0.35	0.35
Limestone	1.29	1.85	1.29	1.85	1.85	1.28	1.84	1.28	1.84	1.84
Salt	0.40	0.40	0.40	0.40	0.40	0.53	0.53	0.53	0.53	0.53
Premix	0.23^3^	0.23^3^	0.28^4^	0.28^4^	0.29^5^	0.18^3^	0.18^3^	0.20^4^	0.20^4^	0.22^5^
*Calculated composition, g/kg*										
DM, %	87.4	87.5	87.3	87.4	87.4	86.1	86.3	86.1	86.3	86.3
P	4.46	4.44	4.46	4.44	4.45	3.54	3.53	3.55	3.53	3.55
Digestible P	2.74	2.74	2.75	2.74	2.75	2.13	2.13	2.14	2.13	2.14
Ca	6.85	8.99	6.85	8.99	8.99	6.42	8.56	6.42	8.56	8.56
Vitamin D3, IU/kg	856	856			2,000	856	856			2,000
25-OH-D3_,_ μg/kg^6^			50.0	50.0				50.0	50.0	
MJ ME^7^	12.3	12.3	12.3	12.3	12.3	11.9	12.0	12.0	11.9	11.9
MJ NE^7^	8.97	8.97	8.97	8.97	8.96	8.74	8.75	8.76	8.75	8.75
CP, %	12.4	12.4	12.4	12.4	12.5	12.0	12.0	12.1	12.0	12.0
Fat	4.01	4.28	4.02	4.28	4.31	5.80	6.10	5.80	6.10	6.10
Ash	4.63	5.17	4.58	5.13	5.15	4.60	5.20	4.60	5.20	5.20
SID^8^ CP	101	101	101	101	101	95.6	95.9	96.5	96.0	96.2
SID^8^ Lys	6.74	6.74	6.74	6.74	6.74	4.28	4.34	4.35	4.35	4.33
SID^8^ Met	2.01	2.00	2.01	2.00	2.01	1.56	1.56	1.57	1.56	1.56
SID^8^ Thr	4.26	4.25	4.26	4.25	4.26	3.15	3.18	3.19	3.18	3.19
SID^8^ Trp	1.36	1.35	1.36	1.35	1.36	1.24	1.25	1.26	1.25	1.25

^1^Dietary treatment from 32 to 100 kg body weight: ACaAD3: 6.85 g calcium/kg and 856 IU/kg vitamin D3; HCaAD3: 8.99 g Ca/kg and 856 IU/kg vitamin D3; ACaHHyD: 6.85 g Ca/kg and 50 μg/kg 25-hydroxyvitamin D3; HCaHHyD: 8.99 g Ca/kg and 50 μg/kg 25-hydroxyvitamin D3; HCaHD3: 8.99 g Ca/kg and 2,000 IU/kg vitamin D3.

^2^Dietary treatment from 100 to 180 kg body weight: ACaAD3: 6.42 g Ca/kg and 856 IU/kg vitamin D3; HCaAD3: 8.56 g calcium/kg and 856 IU/kg vitamin D3; ACaHHyD: 6.42 g Ca/kg and 50 μg/kg 25-hydroxyvitamin D3; HCaHHyD: 8.56 g calcium/kg and 50 μg/kg 25-hydroxyvitamin D3; HCaHD3: 8.56 g calcium/kg and 2,000 IU/kg vitamin D3.

^3^Supplied per kilogram of diet: 8,560 IU vitamin A; 856 IU vitamin D_3_; 107 mg DL-alpha-tocopherol; 2.14 mg vitamin B1; 5.35 mg vitamin B2; 3.21 mg vitamin B6; 0.02 mg vitamin B12; 16.05 mg D-pantothenic acid; 21.40 mg niacin; 1.60 mg folic acid; 0.43 mg biotin; 85.60 mg iron (FeSo_4_); 13.91 mg copper (CuSO_4_); 42.80 mg manganese (MnO); 0.21 mg iodine (Ca(IO_3_)_2_); 0.37 mg selenium (Na_2_SeO_3_). Furthermore, Ronozyme HiPhos GT provided 1,500 phytase activity (FTU) per kg of diet (dsm-firmenich Animal Nutrition and Health, Basel, Switzerland).

^4^Supplied per kilogram of diet: 8,560 IU vitamin A; 50 μg/kg 25-hydroxyvitamin D_3_ (Hy-D, dsm-firmenich Animal Nutrition and Health, Basel, Switzerland); 107 mg DL-alpha-tocopherol; 2.14 mg vitamin B1; 5.35 mg vitamin B2; 3.21 mg vitamin B6; 0.02 mg vitamin B12; 16.05 mg D-pantothenic acid; 21.40 mg niacin; 1.60 mg folic acid; 0.43 mg biotin; 85.60 mg iron (FeSo_4_); 13.91 mg copper (CuSO_4_); 42.80 mg manganese (MnO); 0.21 mg iodine (Ca(IO_3_)_2_); 0.37 mg selenium (Na_2_SeO_3_). Furthermore, Ronozyme HiPhos GT provided 1,500 phytase activity (FTU) per kg of diet (dsm-firmenich Animal Nutrition and Health, Basel, Switzerland).

^5^Supplied per kilogram of diet: 8,560 IU vitamin A; 2,000 IU vitamin D_3_; 107 mg DL-alpha-tocopherol; 2.14 mg vitamin B1; 5.35 mg vitamin B2; 3.21 mg vitamin B6; 0.02 mg vitamin B12; 16.05 mg D-pantothenic acid; 21.40 mg niacin; 1.60 mg folic acid; 0.43 mg biotin; 85.60 mg iron (FeSo_4_); 13.91 mg copper (CuSO_4_); 42.80 mg manganese (MnO); 0.21 mg iodine (Ca(IO_3_)_2_); 0.37 mg selenium (Na_2_SeO_3_). Furthermore, Ronozyme HiPhos GT provided 1,500 phytase activity (FTU) per kg of diet (dsm-firmenich Animal Nutrition and Health, Basel, Switzerland).

^6^25-OH-D3: 25-hydroxyvitamin D3 (dsm-firmenich Animal Nutrition and Health, Basel, Switzerland).

^7^Metabolizable energy (ME) and net energy (NE) were calculated in WinOpti.net (AgroVision Apeldoorn, The Netherlands), according to equations from EvaPig (2020).

^8^SID = standardized ileal digestible.

Within each BW interval, diets were formulated to contain identical levels of all other nutrients according to Danish feeding standards except for Ca, vitamin D3, and 25-hydroxyvitamin D3, which were included at levels exceeding the Danish feeding standards. The gilts were fed 5 different experimental diets in a 2 × 2 factorial design with a fifth diet added alongside. Two Ca levels (6.85/6.42 [adequate; **ACa**] or 8.99/8.56 [high; **HCa**] g/kg were therefore combined with 2 vitamin D sources (856 [Danish feeding standards; adequate; **AD3**] IU/kg vitamin D3 or 50 [high; **HHyD**] μg/kg 25-hydroxyvitamin D3) to create ACaAD3, ACaHHyD, HCaAD3, and HCaHHyD diets. The fifth diet combined HCa and 2,000 IU/kg vitamin D3 (high; **HD3**) to create the **HCaHD3** diet. The values 6.85 and 6.42 g Ca/kg correspond to adequate level, while 8.99 and 8.56 g/kg correspond to a high Ca supply for gilts weighing 32 to 100 and 100 to 180 kg BW, respectively. Adequate level and high levels correspond to 100% and 133% of Ca, and 100% and 234% of vitamin D3/25-hydroxyvitamin D3 according to the Danish feeding standards (Tybirk et al., 2021). The 50 μg/kg 25-hydroxyvitamin D3 is equal to a dose of 2,000 IU/kg. Apart from the variations in Ca and vitamin D (vitamin D3 or 25-hydroxyvitamin D3), the gilts were fed similar levels of other nutrients from 32 kg BW until they reached a slaughter BW of either 100 or 180 kg.

The gilts were fed via an electronic feeding station installed in each pen (COMPIDENT MLP2, Schauer Agrotronic GmbH, Prambachkirchen, Austria) using a radio-frequency identification animal ear tag (Merko M21 FDX + M2, Allflex, Lemvig, Denmark) for individual gilt identification to control the daily maximum feed allowance based on the feeding plan. Since the gilts were fed a restricted diet and since dietary P was not below the requirement in this study, the current feeding strategy did not allow us to evaluate the potential negative effect of excess Ca (Merriman et al., 2017) on voluntary feed intake of the gilts. When the gilts were introduced to the pen, the initial daily feeding level for each gilt was determined based on their initial BW rather than their age. The remaining feeding level during the experimental period gradually increased according to the present feeding curve as illustrated in Supplementary Table 1 until gilts reached 100 kg BW. The gilts were subsequently fed at constant feeding level but with reduced lysine. Irrespective of the dietary treatments, all gilts were fed using the same feeding curve during the rearing period. At each visit to the feeding station, the diet was dispensed in 100 g portion once the gilt had placed her head down in the feed trough. When the gilt had finished eating the first dispensed portion and raised her head, the automatic electronic system closed the feed trough and weighed the feed trough, a process lasting 10 to 15 s. If the gilt remained in the feeder, the feeder dispensed the second portion of the feed, and this cycle would be repeated until the gilt left the feeding station. When the gilt had finished feeding and moved out of the feeding station, the electronic feeding system automatically recorded the feed intake of the gilt. If there was any feed remained in the feed trough from the previous gilt’s visit when the next gilt entered the feeding station, the system would automatically take this residual feed into account in the next gilt’s intake recording. Although the gilts had unlimited access to the feeding station throughout the day, the feed dispenser dispensed the feed only until the daily allowance was reached.

### Feed manufacturing and sampling

Three different premixes were produced to accommodate for the 2 levels of vitamin D3 and 25-hydroxyvitamin D3 that were included in the diets for 32 to 100 and 100 to 180 kg BW gilts. Before and during the production of each batch of the premixes, a rinse batch containing 20 kg of benzoic acid was used to clean the production line, reducing the potential risk of contamination from the previous premix production. All premixes were produced by Vilomix (Vilomix Denmark A/S, Lime, Denmark). Each premix was weighed manually and added to the mixer via a special conveyor system when producing the compound feed used as an experimental diet. The main experimental diets were produced by Danish Agro (Vrå, Denmark). To avoid prolonged storage of the composed diet, the diets were produced twice during each of the experimental periods. This is applied to gilts weighing both 32 to 100 and 100 to 180 kg BW, with a batch size of 2,000 kg. At each feed production stage, a standard gestation diet was mixed before the production of the actual experimental diets. The diets for ACaAD3, HCaAD3, and HCaHD3 groups were then produced in consecutive batches, followed by another production of the standard gestation diet before production of the ACaHHyD and HCaHHyD diets. In each batch of feed production, 20 kg out of the total amount of soybean meal was retained and added as the last feedstuff in the special conveyor system as a rinse batch. During the feed production, 20 kg of each diet was sampled and split into 4 subsamples using a 32-slot riffle sample divider (Pfeuffer GmbH, Kitzingen, Germany). Furthermore, a 5th sample of each dietary treatment was sampled and analyzed after delivery to confirm that the feed had been delivered in the correct silos.

### Recordings and sampling

Gilts were weighed fortnightly from the start to the end of the experimental period using a walk-in scale (Bjerringbro Vægte ApS, Bjerringbro, Denmark). Blood and urine samples were also collected from all gilts shortly before the first round of slaughter at 100 kg BW, and the last samples were collected shortly before the second round of slaughter at 180 kg BW. At each sampling BW, a 9-mL blood sample was collected from each gilt in a 10-mL lithium heparin vacutainer tube (NH Sodium Heparin; Greiner Bio One International GmbH, Kremsmünster, Austria) by puncturing the jugular vein of the gilts. The vacutainers were inverted 10 times to prevent micro-coagulation of the blood samples and were then placed on ice until centrifugation. The blood samples were centrifuged at 2,000 × *g* for 10 min at 4 °C and the resulting plasma aliquots were then harvested in 1.5-mL tubes and stored at −80 °C until analysis. Urine samples were collected in the morning between 0800 and 0900 hours during voluntary urination without using a urinary catheter. Urine samples were collected directly from the gilts during the midpoint of voluntary urination and 50 to 300 mL urine samples were collected into a 700 mL plastic bucket with a long handle. Subsequently, a subsample of 2.5 mL urine was pipetted into a 5-mL Sarstedt CryoPure tube (Hounisen Laboratorieudstyr A/S, Stilling, Denmark) and stored at −20 °C until analysis.

Lameness assessment involved evaluation based on a lameness score and was carried out by a skilled technician using a 4-point scale based on the criteria proposed by Bonde et al. (2004). A description of these criteria is presented in **Table 2**. The assessment procedure entailed temporarily removing the gilts from their pens, allowing them to walk freely in a corridor for 3 min, and subsequently evaluating their lameness. The lameness score was conducted at 80 and 100 kg BW for all gilts and at 140 and 180 kg BW for gilts slaughtered at 180 kg BW. It was consistently carried out by the same technician across all these time points. Furthermore, an experienced technician from Breeding and Genetics at the Danish Agriculture and Food Council assessed the selection rate of gilts to determine whether the gilts met the quality criteria of DanBred gilts. The selection rate was performed shortly before the slaughter day at 100 kg BW (all gilts) and at 180 kg BW for those slaughtered at 180 kg BW. The evaluation criteria for the selection rate included conformation of leg, back, and also gait. Consequently, individual gilts were assigned a binary outcome (yes/no), indicating whether the gilts possessed the necessary qualities to be selected as a breeding animal.

**Table 2.** Description of lameness score assessment criteria in gilts

Score	Category	Description of the criteria
1	No lameness	There is no visible sign of lameness
2	Mild lameness	The gilts will step frequently while standing
3	Moderate lameness	Characterized by attempts to alleviate limb discomfort and uneven gait
4	Severe lameness	Characterized by the inability of the gilts to stand

### Slaughter and bone sampling

Both at 100 and 180 kg slaughter BW, the left front leg of the gilt was carefully removed and sent to the Veterinary Laboratory (Danish Agriculture and Food Council, Kjellerup, Denmark) for subsequent processing. In the laboratory, the 2^nd^ and 3^rd^ metacarpal bones were carefully dissected and separated from the surrounding soft tissue, ensuring the periosteum remained intact and undamaged. The metacarpal bone was used in this study because a previous study had shown a strong correlation (*r* > 0.95) between ash in the metacarpals and total body bone ash concentration (Lee et al., 2021). Following the soft tissue removal, the third metacarpal bone was stored at −20 °C until it was later analyzed for bone length, thickness, and mineral compositions. Meanwhile, the second metacarpal bone was wrapped in gauze, sprayed with an isotonic saline solution containing peptone to stabilize its pH and to prevent the periosteum from drying out, placed in a plastic container with a lid, and stored at −20 °C for later assessment of bone-breaking strength. The bone samples were thawed by defrosting them overnight for the next day’s measurements. The second metacarpal bone was unwrapped from the gauze and immediately placed on a Mecmesin MultiTest 2.5-i-Single Column Force Tester (Kyocera Unimerco Tooling, Sunds, Denmark) to measure the bone-breaking strength. The bone was placed on a flat custom-made probe adjusted to 10 mm from the center on each side. Compression force was applied with 2,500 N force gauges in the middle of the bone length with a compression speed of 50 mm/min. The bone-breaking force was recorded when the first breaking point was detected. The third metacarpal bone was autoclaved for 20 min at 121 °C to aid soft tissue removal. The soft outer tissue was removed from each bone, and the bone weight, length, and thickness were measured immediately. The bone length and thickness were measured using a digital Vernier caliper with a carbide measuring surface (Digital skydelære 150 mm, Diesella A/S, Kolding, Denmark). The bone length measurement represents the longest distance between the proximal and distal points of the metacarpal bone, while the bone thickness measurement represents the narrowest point along the shaft of each metacarpal bone. The third metacarpal bone was subsequently analyzed for total ash, Ca, and P compositions.

### Chemical analysis

All chemical analyses on the diets were performed in duplicate. The dry matter content was determined by drying to a constant weight at 103 °C for 20 h in a forced air oven. Analyses for crude protein, minerals, and amino acids (EC 64/1998 and EC 152/2009) were performed in accordance with the European Commission Directives according to the *Official Journal of the European Union* (European-Commission, 2009) at a commercial laboratory (Eurofins Steins Laboratory A/S, Vejen, Denmark). Concentrations of vitamin D3 and 25-hydroxyvitamin D3 were analyzed in the Bio-analytics laboratory of dsm-firmenich Animal Nutrition & Health (Kaiseraugst, Switzerland). The concentration of the vitamin D3 was determined using HPLC/MS/MS as described by Schadt et al. (2019), and the concentration of 25-hydroxyvitamin D3 was determined using a patented method including extraction with tert-butyl methyl ether and subsequently using HPLC/MS as described by Denu et al. (2008).

The Ca and P contents in the metacarpal bone were analyzed following the destruction of organic matter. The bones were cut in half to fit better in the crucible and to achieve as large a surface area as possible for better and faster drying/ashing. Samples of the whole bones were ashed in silica crucibles at 525 °C for 6 h. After cooling, the ashes were ground using an IKA mill (starting at 5,000 rpm and increasing to a maximum of 25,000 rpm), and 0.25 g of the ground ash samples were subsequently transferred to a sample tube and dissolved in 4 mL concentrated HNO_3_. The samples were then transferred to a Greiner tube with Milli-Q water and filled up to approximately 50 mL. Finally, the Ca and P contents in the bone were analyzed on an iCAP TQ ICP-MS (Inductively Coupled Plasma Mass Spectrometer) equipped with a MicroMist DC nebulizer, a Quartz cyclonic spray chamber operated at 2.7 °C (Thermo Fisher Scientific, Bremen, Germany) and a CETAC auto sampler model ASX 560. The instrument settings were forward power 1,550 W, plasma gas (Ar) 14 L/min, nebulizer gas (Ar) 1.0 L/min, and auxiliary gas (Ar) 0.8 L/min. The sample uptake was approximately 0.4 mL/min. Data were collected using QtegraTM version 2.10.9.3324.131 (Thermo Fisher Scientific). The minerals were analyzed in kinetic energy discrimination mode and ^43^Ca and ^31^P isotopes were used for quantification.

Concentrations of Ca, inorganic P, and total alkaline phosphatase (**TAP**) activity in plasma, and Ca and P in urine were determined according to standard procedures (Siemens Diagnostics Clinical Methods for ADVIA 1800). Porcine bone-specific alkaline phosphatase (**BAP**), and C-terminal cross-linking telopeptide of type I collagen (**CTX-I**) were determined by Elisa assays (BioSite EDKX-SZ14LY-96 and EKX-4JAOTA-96, respectively). Pig osteocalcin was determined by Elisa assay (OKEH00463, Aviva Systems Biology, Corp. San Diego, CA 92,121). Recommendations given by the manufacturers were followed for all Elisa assays.

Plasma concentrations of vitamin D3 and its metabolites were analyzed at the Bio-analytics lab of dsm-firmenich Animal Nutrition and Health using an internally validated method previously described by Rider et al. (2023). In brief, an internal standard was added to an aliquot of plasma, and vitamin D3 and its main known metabolites (24,25-dihydroxy-vitamin D3 and 25-hydroxy-vitamin D3) were subsequently extracted by protein precipitation with acetonitrile. After centrifugation and filtration, the supernatant was evaporated, and the residue was reconstituted with a methanol-acetonitrile-water solution. The analysis was performed by reverse phase chromatography (UHPLC, 1290 Agilent Infinity II LC System, Agilent, Santa Clare, CA) coupled with MS detection (SCIEX API4000, Sciex, Framingham, MA, or Agilent 6495QQQ, Agilent). Quantification was done by applying dedicated external calibrations (using deuterated internal standards).

### Statistical analyses

All statistical analyses were performed using SAS (SAS 9.3, SAS Institute Inc., Cary, NC). The individual gilt was considered the experimental unit. Initial age and BW, average age and weight at 100 and 180 kg BW, ADG, and feed conversion ratio, plasma concentrations of minerals, vitamin D3 and its main metabolites, bone turnover biomarkers, bone weight, length, strength, thickness, mineral compositions, and urine concentration of Ca and P were analyzed using the MIXED procedure of SAS including dietary treatments and boar as fixed effects. The gilt selection rate and lameness score were analyzed using the GLIMMIX procedure of SAS (assuming binary distribution) including dietary treatments and genetic boar as fixed effects. In both models, initial BW was included as a covariate. Pearsons’ correlation coefficient of ADG and plasma concentration of 25-hydroxyvitamin D3 with lameness, selection rate, and bone parameters were analyzed using CORR procedure of SAS.

Besides testing the overall effect of the 5 dietary treatments, by omitting the HCaHD3 dietary group, the remaining 4 dietary groups were contrasted to test the effect of excess Ca irrespective of vitamin D3 source and level by comparing HCaAD3 and HCaHHyD with ACaAD3 and ACaHHyD. Additionally, the effect of dietary vitamin D3 source and level irrespective of Ca level was tested by comparing ACaAD3 and HCaAD3 with ACaHHyD and HCaHHyD. Furthermore, all models were adjusted by excluding the HCaHD3 group and subsequently analyzed for potential interactions between Ca and vitamin D using factorial analysis. The data are reported as LSMEANS with the largest SEM; except in figures where LSMEANS are presented with their corresponding SE. Statistical difference was accepted at *P *≤ 0.05, whereas *P* ≤ 0.10 was considered a tendency.

## Results

### Compositions of the experimental diets

The Ca content in the diets for gilts weighing 32 to 100 kg BW varied between −4.1% and +1.6% of the expected values (**Table 3**). For gilts weighing 100 to 180 kg BW, the analyzed compositions of Ca in the diets showed a slightly larger deviation, varied between −7.6% and −2.7%. The 25-hydroxyvitamin D3 content in diets for gilts weighing 32 to 100 and 100 to 180 kg BW varied between −4.6% and −10% of the expected values, respectively. In the high inclusion level diet for 32 to 100 kg BW gilts, the analyzed vitamin D3 composition exceeded the expected value by 12%. The analyzed content of phytase was close to the expected values, although for gilts weighing 100-180 kg BW the ACaHHyD and HCaHHyD diets contained 14.6% and 24.3% less phytase activity than expected, respectively.

**Table 3.** Analyzed chemical compositions (as-fed) of the dietary treatments used for feeding gilts weighing 32 to 180 kg body weight

Item	32 to 100 kg body weight^1^					100 to 180 kg body weight^2^				
	ACaAD3	HCaAD3	ACaHHyD	HCaHHyD	HCaHD3	ACaAD3	HCaAD3	ACaHHyD	HCaHHyD	HCaHD3
DM, %	87.3	87.3	87.2	87.1	87.0	86.3	86.2	86.3	86.2	86.4
CP, %	12.9	12.8	12.9	12.9	13.0	11.8	11.9	11.9	11.8	12.1
Phytase, FTU/kg^3^	1,567	1,558	1,337	1,640	1,420	1,479	1,281	1,136	1,240	1,465
P, g/kg	4.60	4.57	4.61	4.56	4.61	3.34	3.42	3.45	3.34	3.37
Ca, g/kg	6.83	8.62	6.96	8.78	8.66	5.93	7.92	6.22	8.32	8.33
Mg, g/kg	1.42	1.45	1.44	1.46	1.50	1.27	1.29	1.22	1.19	1.26
Zn, mg/kg	125	124	124	122	123	116	114	122	115	117
Cu, mg/kg	16	15	15	15	15	17	16	15	16	16
Mn, mg/kg	62	62	63	62	62	61	60	62	62	63
Vitamin D3, IU/kg	850	850			2,240	882	882			1,949
25-OH-D3, μg/kg^4^			47.7	47.7				44.8	44.8	
Lys, g/kg	7.57	7.63	7.38	7.62	7.53	5.73	5.67	5.80	5.59	5.77
Met, g/kg	2.18	2.10	2.19	2.12	2.11	1.95	1.85	1.96	1.90	1.88
Cys, g/kg	2.56	2.52	2.55	2.54	2.58	2.49	2.47	2.49	2.42	2.45
Thr, g/kg	4.99	4.95	5.01	5.05	5.06	4.28	4.24	4.30	4.13	4.40

^1^Dietary treatment from 32 to 100 kg body weight: ACaAD3: 6.85 g calcium/kg and 856 IU/kg vitamin D3; HCaAD3: 8.99 g Ca/kg and 856 IU/kg vitamin D3; ACaHHyD: 6.85 g Ca/kg and 50 μg/kg 25-hydroxyvitamin D3; HCaHHyD: 8.99 g Ca/kg and 50 μg/kg 25-hydroxyvitamin D3; HCaHD3: 8.99 g Ca/kg and 2,000 IU/kg vitamin D3.

^2^Dietary treatment from 100 to 180 kg body weight: ACaAD3: 6.42 g Ca/kg and 856 IU/kg vitamin D3; HCaAD3: 8.56 g calcium/kg and 856 IU/kg vitamin D3; ACaHHyD: 6.42 g Ca/kg and 50 μg/kg 25-hydroxyvitamin D3; HCaHHyD: 8.56 g calcium/kg and 50 μg/kg 25-hydroxyvitamin D3; HCaHD3: 8.56 g calcium/kg and 2,000 IU/kg vitamin D3.

^3^FTU = phytase units.

^4^25-OH-D3: 25-hydroxyvitamin D3 (Hy-D, dsm-firmenich Animal Nutritional and Health, Basel, Switzerland).

### Production performance

The gilts were similar in their initial BW and age across the 5 dietary treatments (**Table 4**), indicating effective randomization. The results showed no significant differences in ADG (*P* = 0.34 and *P* = 0.79) and feed efficiency (*P* = 0.16 and *P* = 0.89) among gilts weighing between 32-100 and 100-180 kg BW, respectively. However, it is noteworthy that the ADG and feed efficiency decreased numerically as the gilts aged. Despite their similar initial BW, ADG, and feed efficiency, gilts fed the ACaHHyD diet and slaughtered at 100 kg BW unexpectedly showed a lower final BW (*P* = 0.03) compared with the other groups.

**Table 4.** The effect of different levels of dietary calcium, and sources and level of D3 on performances of gilts fed the 5 dietary treatments during the growth phase (32 to 180 kg BW)

Item	Dietary treatment^1,2^						
	ACaAD3	HCaAD3	ACaHHyD	HCaHHyD	HCaHD3	SEM^3^	*P*-value
*All gilts 32 to 180 kg BW, n*	36	35	35	38	37		
Initial BW, kg	32.3	32.6	32.0	32.5	32.7	0.94	0.99
Initial age, d	80.2	79.9	80.5	80.6	80.3	0.42	0.81
*All gilts 32 to 100 kg BW*							
Average daily gain, g/d	772	777	757	788	760	17.9	0.47
Feed:gain ratio	2.83	2.82	2.86	2.76	2.85	0.05	0.48
*Gilts slaughtered at 100 kg BW, n*	18	17	19	20	19		
BW at slaughter, kg	106^a^	108^a^	98.0^b^	107^a^	102^a,b^	2.61	0.035
Age at slaughter, d	171	171	172	172	171	1.72	0.99
Average daily gain, g/d	776	800	743	792	765	23.8	0.34
Feed:gain ratio	2.80	2.73	2.91	2.72	2.81	0.06	0.16
*Gilts slaughtered at 180 kg BW, n*	18	18	16	18	18		
BW at slaughter, kg	183	183	181	181	180	3.22	0.96
Age at slaughter, d	291	291	292	292	292	1.76	0.99
Average daily gain, g/d	686	678	665	670	662	15.7	0.79
Feed:gain ratio	3.83	3.88	3.88	3.92	3.80	0.11	0.89

^a,b^Means within a row with different superscripts differ (*P *< 0.05).

^1^Dietary treatment from 32 to 100 kg BW: ACaAD3: 6.85 g calcium/kg and 856 IU/kg vitamin D3; HCaAD3: 8.99 g Ca/kg and 856 IU/kg vitamin D3; ACaHHyD: 6.85 g Ca/kg and 50 μg/kg 25-hydroxyvitamin D3; HCaHHyD: 8.99 g Ca/kg and 50 μg/kg 25-hydroxyvitamin D3; HCaHD3: 8.99 g Ca/kg and 2,000 IU/kg vitamin D3.

^2^Dietary treatments from 100 to 180 kg BW: ACaAD3: 6.42 g Ca/kg and 856 IU/kg vitamin D3; HCaAD3: 8.56 g calcium/kg and 856 IU/kg vitamin D3; ACaHHyD: 6.42 g Ca/kg and 50 μg/kg 25-hydroxyvitamin D3; HCaHHyD: 8.56 g calcium/kg and 50 μg/kg 25-hydroxyvitamin D3; HCaHD3: 8.56 g calcium/kg and 2,000 IU/kg vitamin D3.

^3^The largest standard error of means for the mixed procedure.

### Plasma metabolites and urinary concentration of Ca and P

The dietary treatments had no impact on the plasma concentration of Ca, irrespective of sampling BW and Ca levels. However, high dietary Ca reduced the plasma concentration of P, although it was not exclusively consistent across the 2 slaughter BW (**Table 5**). Thus, in plasma samples collected at 100 kg BW, gilts fed the ACaHHyD diet had a greater concentration of P than those fed the HCaAD3 (*P* = 0.01) and HCaHHyD (*P* = 0.005) diets. However, in plasma samples collected at 180 kg BW, gilts fed HCaAD3 diet had a lower concentration of P than those fed ACaAD3 (*P* < 0.001), ACaHHyD (*P* < 0.001), and HCaHHyD (*P* = 0.01) diets.

**Table 5.** The effect of different levels of dietary calcium, and sources and level of D3 on plasma levels of calcium, phosphorus, vitamin D3, vitamin D3 metabolites, plasma bone turnover biomarkers, and urine concentrations of calcium and phosphorus in gilts fed the dietary treatments from 32 to 80 kg BW and plasma samples were collected immediately before slaughter at both 100 and 180 kg BW

	Dietary treatments (Trt) ^1,2^						*P*-values^4^		
	ACaAD3	HCaAD3	ACaHHyD	HCaHHyD	HCaHD3	SEM^3^	Trt	Ca level	D3 source and level
*At 100 kg BW, n* ^5^	37	37	36	39	37				
*Plasma*									
Ca, mM	2.74	2.78	2.75	2.74	2.79	0.027	0.56	0.57	0.81
P, mM	2.85^ab^	2.79^b^	2.92^a^	2.77^b^	2.83^ab^	0.049	0.03	0.005	0.19
Vitamin D3, ng/mL	2.30^b^	2.08^b^	0.25^c^	0.25^c^	5.20^a^	0.103	<0.001	0.27	<0.001
25-OH-D3, ng/mL	18.2^c^	17.3^c^	92.5^a^	89.8^a^	24.9^b^	2.04	<0.001	0.34	<0.001
24,25(OH)_2_-D3, ng/mL	5.77^c^	5.44^c^	36.9^a^	35.8^a^	8.16^b^	0.787	<0.001	0.37	<0.001
Osteocalcin, ng/mL	348	368	390	370	387	18.9	0.38	1.00	0.08
TAP, U/L^6^	180	190	187	182	185	8.84	0.91	0.80	0.57
BAP, ng/mL^7^	5.58	4.69	4.76	5.49	4.77	0.299	0.06	0.78	0.05
CTX-I, ng/mL^8^	9.01	8.26	8.73	8.33	8.33	0.539	0.83	0.28	0.71
*Urine*									
Ca, mM	1.13^c^	2.51^ab^	1.70^bc^	2.27^ab^	2.74^a^	0.359	0.004	0.003	0.22
P, mM	3.62^a^	1.03^b^	5.12^a^	1.80^b^	1.04^b^	0.555	<0.001	<0.001	0.07
*At 180 kg BW, n*	18	18	16	18	18				
*Plasma*									
Ca, mM	2.67	2.64	2.63	2.66	2.63	0.035	0.86	0.99	0.35
P, mM	2.48^a^	2.23^b^	2.47^a^	2.38^a^	2.35^ab^	0.052	0.002	0.0009	0.86
Vitamin D3, ng/mL	2.48^b^	2.11^c^	0.24^d^	0.24^d^	5.75^a^	0.121	<0.001	0.10	<0.001
25-OH-D3, ng/mL	19.5^c^	17.9^c^	80.4^a^	88.5^a^	29.9^b^	2.59	<0.001	0.76	<0.001
24,25(OH)_2_-D3, ng/mL	8.09^c^	7.94^c^	42.9^a^	44.1^a^	12.9^b^	1.40	<0.001	0.70	<0.001
Osteocalcin, ng/mL	336	363	327	368	336	19.7	0.43	0.06	0.72
TAP, U/L^6^	117	113	124	125	127	9.55	0.68	0.84	0.63
BAP, ng/mL^7^	3.63^b^	3.40^b^	3.28^b^	3.04^b^	4.66^a^	0.36	0.02	0.53	0.51
CTX-I, ng/mL^8^	8.3	9.2	8.1	10.2	8.0	1.4	0.59	0.19	0.83
*Urine*									
Ca, mM	2.11^b^	4.31^ab^	4.43^ab^	6.46^a^	4.30^ab^	1.154	0.05	0.05	0.11
P, mM	1.86^a^	0.28^b^	1.30^ab^	1.05^ab^	0.35^b^	0.492	0.04	0.04	0.36

^a,b,c,d^Means within a row with different superscript differ (*P *< 0.05).

^1^Dietary treatment from 32 to 100 kg BW: ACaAD3: 6.85 g calcium/kg and 856 IU/kg vitamin D3; HCaAD3: 8.99 g Ca/kg and 856 IU/kg vitamin D3; ACaHHyD: 6.85 g Ca/kg and 50 μg/kg 25-hydroxyvitamin D3; HCaHHyD: 8.99 g Ca/kg and 50 μg/kg 25-hydroxyvitamin D3; HCaHD3: 8.99 g Ca/kg and 2,000 IU/kg vitamin D3.

^2^Dietary treatments from 100 to 180 kg BW: ACaAD3: 6.42 g Ca/kg and 856 IU/kg vitamin D3; HCaAD3: 8.56 g calcium/kg and 856 IU/kg vitamin D3; ACaHHyD: 6.42 g Ca/kg and 50 μg/kg 25-hydroxyvitamin D3; HCaHHyD: 8.56 g calcium/kg and 50 μg/kg 25-hydroxyvitamin D3; HCaHD3: 8.56 g calcium/kg and 2,000 IU/kg vitamin D3.

^3^The largest SEM values within a group are reported.

^4^Trt: the effect of the overall dietary treatments; Ca: the effect of dietary levels of Ca regardless of vitamin D3 sources and level by comparing ACaAD3 and ACaHHyD with HCaAD3, HCaHHyD; D3 source and level: the effect of vitamin D3 source and level regardless of dietary Ca level by comparing ACaAD3 and HCaAD3 with ACaHHyD and HCaHHyD.

^5^Plasma and urine samples were collected from all gilts at 100 kg BW.

^6^Total alkaline phosphatase.

^7^Bone-specific alkaline phosphatase.

^8^CTX-I = C-terminal telopeptides of type I collagen.

The urinary concentration of Ca at 100 kg BW was lowest (1.13 mM) in gilts fed ACaAD3, intermediate (1.70 mM) in gilts fed ACaHHyD, and greatest (2.27 to 2.74 mM) in the remaining groups (*P* < 0.001; mean comparison). In urine samples collected at 180 kg BW, gilts fed the HCaHHyD diet had greater urinary concentrations of Ca than gilts fed the ACaAD3 diet (*P* = 0.003). Moreover, gilts fed low Ca diets, regardless of the vitamin D3 sources, had greater concentrations of P in the urine at both 100 (*P* < 0.001) and 180 (*P* = 0.04) kg BW.

**Figure 1.**
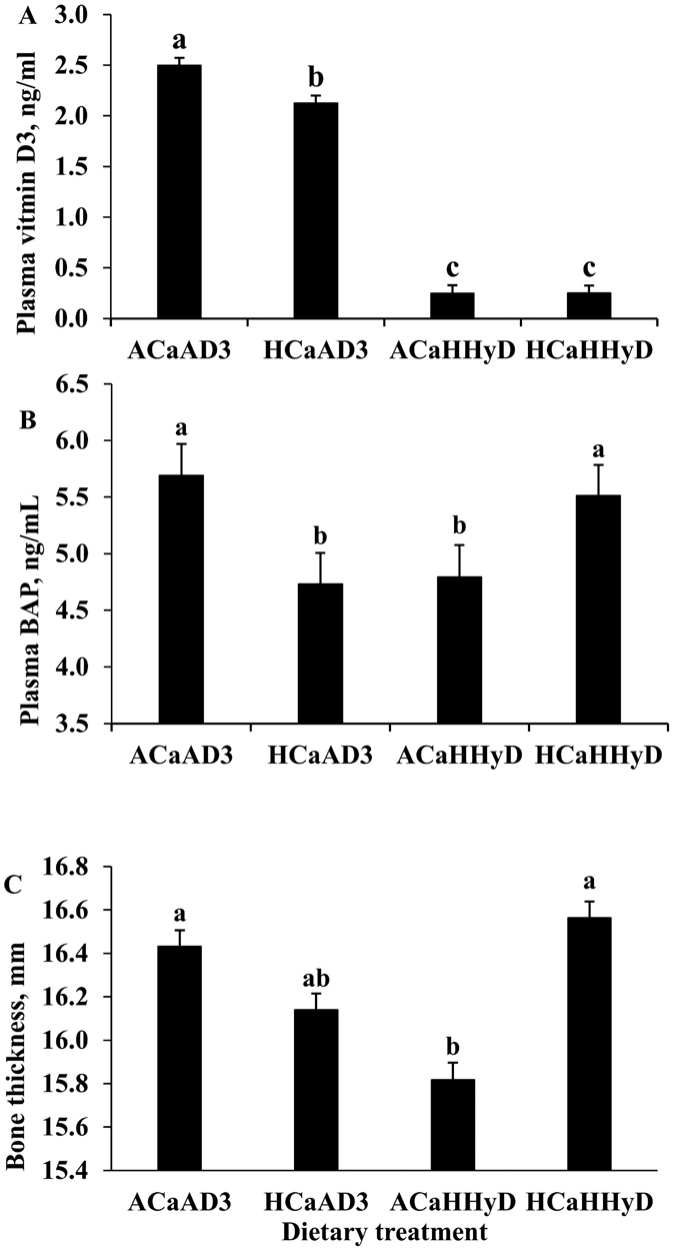
Interactions between dietary calcium and vitamin D for (A) plasma vitamin D3, (B) plasma bone specific alkaline phosphatase (**BAP**), and (C) bone thickness in gilts fed different levels of calcium and vitamin D based dietary treatment from 32 to 180 kg BW. ACaAD3: 6.85 g/kg calcium and 856 IU/kg vitamin D3; HCaAD3: 8.99 g/kg calcium and 856 IU/kg vitamin D3; ACaHHyD: 6.85 g/kg calcium and 50 μg/kg 25-hydroxyvitamin D3; HCaHHyD: 8.99 g/kg calcium and 50 μg/kg 25-hydroxyvitamin D3. Bars with different letter differ (*P* < 0.05). The data is presented as LSMEANS with standard error.

Plasma concentrations of both vitamin D3 and 25-hydroxyvitamin D3 exhibited dose-dependent responses at 100 and 180 kg BW, with supplementation of these components leading to increases in plasma concentrations (*P* < 0.001). An interaction between dietary Ca and vitamin D on plasma concentration of vitamin D3 was observed when the HCaHD3 group was excluded from the model (Fig. 1A). The plasma concentration of vitamin D3 was greatest (5.20 to 5.75 ng/mL) in gilts fed the HCaHD3 diet, intermediate (2.08 to 2.48 ng/mL) in gilts fed ACaAD3 and HCaAD3, and lowest (0.24 to 0.25 ng/mL) in gilts fed ACaHHyD and HCaHHyD diets across the sampling BW (*P* < 0.001; mean comparison). Moreover, the plasma concentration of 25-hydroxyvitamin D3 was greatest (80.4 to 92.5 ng/mL) in gilts fed the ACaHHyD and HCaHHyD diets, intermediate (24.9 to 29.9 ng/mL) in gilts fed HCaHD3 diet, and lowest (17.3 to 19.5 ng/mL) in gilts fed ACaAD3 and HCaHD3 diets across the sampling BW (*P* < 0.001; mean comparison). The plasma concentration of 24,25 dihydroxyvitamin D3 was greatest for gilts fed ACaHHyD and HCaHHyD (35.8 to 44.1 ng/mL), intermediate for gilts fed HCaHD3 (8.16 to 12.9 ng/mL) and lowest for gilts fed ACaAD3 and HCaAD3 (5.44 to 8.09 ng/mL) across the sampling BW (*P* < 0.001; mean comparison). The dietary content of Ca affects neither the plasma concentration of vitamin D3 nor its metabolites at both 100 and 180 kg BW.

In plasma samples collected at 100 kg BW, the concentration of BAP tended to be greater in gilts fed ACaAD3 and HCaHHyD diets than in the remaining groups (*P* = 0.06). Moreover, an interaction between dietary Ca and vitamin D on BAP concentration was observed when the HCaHD3 group was excluded from the model (Fig. 1B). However, in plasma samples collected at 180 kg BW, the concentration of BAP was greater in gilts fed the HCaHD3 diet than in the remaining groups (*P* = 0.02). In general, the plasma concentrations of osteocalcin and CTX-I were consistently similar among the dietary treatments and the sampling BW. However, the activity of TAP and BAP seemed to be more active in plasma samples collected at 100 kg BW than in plasma samples collected at 180 kg BW.

### Bone measurements and mineral compositions

There were no dietary treatment effects on bone weight, length, thickness, dry matter, or Ca and P concentrations regardless of the slaughter BW (**Table 6**). Nevertheless, bone ash content per bone was greater in gilts fed the HCaAD3 diet compared with those fed ACaAD3 and ACaHHyD and slaughtered at 100 kg BW (*P* = 0.02). Additionally, gilts fed the HCaHD3 and HCaAD3 diets had greater bone-breaking strength compared with those fed the ACaAD3, ACaHHyD, and HCaHHyD diets when slaughtered at 100 kg BW (*P* = 0.05). However, an interaction between dietary Ca and vitamin D on bone thickness was observed when the HCaHD3 treatment group was excluded from the main model (Fig. 1C).

**Table 6.** The effect of different levels of dietary calcium, and source and level of vitamin D3 on dimensions, compositions, and breaking strength of metacarpal bone in gilts fed the dietary treatments from 32 to 180 kg BW and slaughtered at either 100 or 180 kg BW

	Dietary treatments (Trt)^1,2^						*P*-value^3^		
	ACaAD3	HCaAD3	ACaHHyD	HCaHHyD	HCaHD3	SEM^4^	Trt	Ca level	D3 source and level
*Slaughtered at 100 kg BW, n*	18	16	19	19	18				
Wet weight, g	17.3	17.3	17.4	17.1	17.2	0.091	0.19	0.06	0.47
Length, mm	60.3	60.5	60.6	60.3	59.6	0.49	0.57	0.97	0.63
Thickness, mm	16.2	16.2	16.0	16.5	16.3	0.184	0.30	0.13	0.37
Breaking strength, N	485^b^	537	481	492	551	35.1	0.05	0.12	0.89
DM, %	87.1	87.4	86.7	88.0	87.8	0.542	0.12	0.05	0.52
Ash, g/bone	6.64^b^	7.07^a^	6.66^b^	6.81^ab^	6.81^ab^	0.102	0.02	0.003	0.88
Ca, g/bone	2.10	2.22	2.08	2.16	2.11	0.073	0.49	0.10	0.79
P, g/bone	0.99	1.05	0.98	1.02	0.99	0.034	0.39	0.07	0.70
*Slaughtered at 180 kg BW, n*	18	18	16	18	18				
Wet weight, g	26.3	26.2	26.1	26.3	26.1	0.113	0.74	0.63	0.26
Length, mm	70.0	69.8	70.2	70.4	70.5	0.607	0.91	0.97	0.75
Thickness, mm	18.0	18.2	18.3	18.2	18.6	0.341	0.73	0.80	0.58
Breaking strength, N	1,025	1,022	923	1,001	1,014	42.7	0.36	0.34	0.08
DM, %	88.4	88.7	89.0	88.4	88.8	0.359	0.70	0.62	0.24
Ash, g/bone	11.5	11.6	11.1	11.3	11.1	0.206	0.26	0.43	0.16
Ca, g/bone	3.25	3.33	3.16	3.28	3.19	0.059	0.21	0.07	0.28
P, g/bone	1.50	1.53	1.47	1.51	1.47	0.028	0.36	0.18	0.42

^a,b^Means within a row with different superscripts differ (*P *< 0.05).

^1^Dietary treatment from 32 to 100 kg BW: ACaAD3: 6.85 g calcium/kg and 856 IU/kg vitamin D3; HCaAD3: 8.99 g Ca/kg and 856 IU/kg vitamin D3; ACaHHyD: 6.85 g Ca/kg and 50 μg/kg 25-hydroxyvitamin D3; HCaHHyD: 8.99 g Ca/kg and 50 μg/kg 25-hydroxyvitamin D3; HCaHD3: 8.99 g Ca/kg and 2,000 IU/kg vitamin D3.

^2^Dietary treatments from 100-180 kg BW: ACaAD3: 6.42 g Ca/kg and 856 IU/kg vitamin D3; HCaAD3: 8.56 g calcium/kg and 856 IU/kg vitamin D3; ACaHHyD: 6.42 g Ca/kg and 50 μg/kg 25-hydroxyvitamin D3; HCaHHyD: 8.56 g calcium/kg and 50 μg/kg 25-hydroxyvitamin D3; HCaHD3: 8.56 g calcium/kg and 2,000 IU/kg vitamin D3.

^3^Trt: the effect of the overall dietary treatments; Ca: the effect of dietary levels of Ca regardless of vitamin D3 sources and level by comparing ACaAD3 and ACaHHyD with HCaAD3 and HCaHHyD; D3 source and level: the effect of vitamin D3 source and level regardless of dietary Ca level by comparing ACaAD3 and HCaAD3 with ACaHHyD and HCaHHyD.

^4^The largest SEM values within a group is reported.

### Selection rate and lameness scores

The selection rate and lameness scores were not impacted by the dietary treatments or the levels of Ca and level and source of vitamin D3 (**Table 7**). At 100 kg BW, a tendency for a lower selection rate (*P* = 0.09) was observed in gilts fed the high Ca diets compared with those fed the adequate Ca diets. Regardless of the dietary treatments, there appeared to be a numerical increase in the odds of lameness in gilts slaughtered at 180 kg BW compared with those slaughtered at 100 kg BW, specifically in the moderate lameness score category.

**Table 7.** The effect of different levels of dietary calcium, and source and level of vitamin D_3_ on lameness score and selection rate in gilts fed the dietary treatments from 32 to 180 kg BW and evaluated at 100 and 180 kg BW

	Dietary treatments (Trt)^1,2^						*P*-value^3^		
	ACaAD3	HCaAD3	ACaHHyD	HCaHHyD	HCaHD3	SEM^4^	Trt	Ca level	D3 source and level
*Slaughtered at 100 kg BW, n*	18	16	19	19	18				
No lameness, %	56	64	58	67	68	8	0.76	0.27	0.89
Mild lameness, %	36	33	36	33	30				
Moderate lameness, %	8	3	6	0	3				
Severe lameness, %	0	0	0	0	0				
Selection rate = yes, %	92	84	92	78	86	6	0.48	0.09	0.83
*Slaughtered at 180 kg BW, n*	17	17	16	18	18				
No lameness, %	47	53	62	44	50	15	0.84	0.66	0.30
Mild lameness, %	24	29	19	39	44				
Moderate lameness, %	29	18	19	17	6				
Severe lameness, %	0	0	0	0	0				
Selection rate = yes, %	72	88	88	78	94	10	0.49	0.80	0.29

^1^Dietary treatment from 32 to 100 kg BW: ACaAD3: 6.85 g calcium/kg and 856 IU/kg vitamin D3; HCaAD3: 8.99 g Ca/kg and 856 IU/kg vitamin D3; ACaHHyD: 6.85 g Ca/kg and 50 μg/kg 25-hydroxyvitamin D3; HCaHHyD: 8.99 g Ca/kg and 50 μg/kg 25-hydroxyvitamin D3; HCaHD3: 8.99 g Ca/kg and 2,000 IU/kg vitamin D3.

^2^Dietary treatments from 100 to 180 kg BW: ACaAD3: 6.42 g Ca/kg and 856 IU/kg vitamin D3; HCaAD3: 8.56 g calcium/kg and 856 IU/kg vitamin D3; ACaHHyD: 6.42 g Ca/kg and 50 μg/kg 25-hydroxyvitamin D3; HCaHHyD: 8.56 g calcium/kg and 50 μg/kg 25-hydroxyvitamin D3; HCaHD3: 8.56 g calcium/kg and 2,000 IU/kg vitamin D3.

^3^Trt: the effect of the overall dietary treatments; Ca: the effect of dietary levels of Ca regardless of vitamin D3 sources and level by comparing ACaAD3 and ACaHHyD with HCaAD3, HCaHHyD; D3 source and level: the effect of vitamin D3 source and level regardless of dietary Ca level by comparing ACaAD_3_ and HCaAD3 with ACaHHyD and HCaHHyD.

^4^The largest SEM values within a group is reported.

### Pearsons’ correlation coefficient

The ADG of gilts weighing 32 to 100 kg BW was positively correlated with bone strength (*r* = 0.37; *P* < 0.001; **Table 8**), whereas it tended to be positively correlated for ADG from 100 to 180 kg BW (*r* = 0.20; *P* = 0.07). The ADG of gilts weighing 100 to 180 kg BW displayed a positive correlation with bone ash content (*r *= 0.24; *P* = 0.02), but negative correlation with the lameness score (*r *= -0.25; *P* = 0.02). The plasma concentration of 25-hydroxyvitamin D3 demonstrated a negative correlation with bone strength in gilts fed a vitamin D3-supplemented diet and slaughtered at 180 kg BW (*r* = −0.34; *P* = 0.02). Conversely, the plasma concentration of 25-hydroxyvitamin D3 showed a negative correlation with lameness (*r* = −0.35; *P* = 0.03) in gilts fed a 25-hydroxyvitamin D3-supplemented diet and slaughtered at 100 kg BW. Interestingly, under the same dietary treatment but slaughtered at 180 kg BW, the plasma concentration of 25-hydroxyvitamin D3 displayed a positive correlation with ash (*r* = 0.37; *P* = 0.03) and P (*r* = 0.34; *P* = 0.05), and tendency with Ca (*r* = 0.30; *P* = 0.08) contents of the bone.

**Table 8.** Pearson correlation coefficients between variables in gilts slaughtered at 100 and 180 kg BW that were fed different levels of dietary calcium, and source and level of vitamin D_3_ and fed the dietary treatments from 32 to 180 kg BW

Items	ADG		Plasma 25-hydroxyvitamin D3^1, 2^			
			Treat 1, 2, and 5		Treat 3 and 4	
	*r*	*P*-value	*r*	*P*-value	*r*	*P*-value
*Gilts slaughtered at 100 kg BW*	*32 to 100 kg BW*					
Lameness	−0.03	0.79	−0.09	0.54	−0.35	0.03
Selection rate	−0.11	0.28	0.06	0.69	−0.05	0.75
Bone strength, N	0.37	<0.001	0.12	0.39	0.07	0.68
Bone ash content, g/bone	0.16	0.13	−0.06	0.67	0.24	0.15
Bone calcium content, g/bone	0.09	0.37	−0.01	0.94	0.12	0.49
Bone phosphorus content, g/bone	0.11	0.31	−0.03	0.85	0.05	0.74
*Gilts slaughtered at 180 kg BW*	*100 to 180 kg BW*					
Lameness	−0.25	0.02	−0.09	0.54	−0.09	0.61
Selection rate	0.16	0.13	0.15	0.28	0.01	0.94
Bone strength, N	0.20	0.07	−0.34	0.02	−0.13	0.45
Bone ash content, g/bone	0.24	0.02	−0.26	0.06	0.37	0.03
Bone calcium content, g/bone	0.14	0.18	−0.18	0.20	0.30	0.08
Bone phosphorus content, g/bone	0.14	0.21	−0.19	0.16	0.34	0.05

^1^Dietary treatment from 32 to 100 kg BW: ACaAD_3_: 6.85 g calcium/kg and 856 IU/kg vitamin D3; HCaAD3: 8.99 g Ca/kg and 856 IU/kg vitamin D3; ACaHHyD: 6.85 g Ca/kg and 50 μg/kg 25-hydroxyvitamin D3; HCaHHyD: 8.99 g Ca/kg and 50 μg/kg 25-hydroxyvitamin D3; HCaHD3: 8.99 g Ca/kg and 2,000 IU/kg vitamin D3.

^2^Dietary treatments from 100-180 kg BW: ACaAD3: 6.42 g Ca/kg and 856 IU/kg vitamin D3; HCaAD3: 8.56 g calcium/kg and 856 IU/kg vitamin D3; ACaHHyD: 6.42 g Ca/kg and 50 μg/kg 25-hydroxyvitamin D3; HCaHHyD: 8.56 g calcium/kg and 50 μg/kg 25-hydroxyvitamin D3; HCaHD3: 8.56 g calcium/kg and 2,000 IU/kg vitamin D3.

## Discussion

### Growth performance

The gilts showed similar growth performance across all dietary treatments, as each diet was optimized to meet the nutrient requirements for growing gilts weighing 30 to 180 kg BW according to Danish nutrient standards (Tybirk et al., 2021). The age-dependent sigmoid-shaped growth curve in gilts, with an inflection point at approximately 90 to 103 kg BW (Danfær and Strathe, 2012), partly explains the observed decline in ADG and feed efficiency as the gilts grow. In support of this, Luo et al. (2015) observed a rapid growth rate in pigs aged between 100 to 190 d compared with the later age until day 250. Nevertheless, the results observed in the present study align with those in previous studies which also showed no impact on ADG and feed efficiency in growing pigs fed varying levels of dietary Ca (Nicodemo et al., 1998; Letourneau-Montminy et al., 2010), vitamin D3 (O’Doherty et al., 2010), or 25-hydroxyvitamin D3 (Regassa et al., 2015). In contrast, Zhai et al. (2023) observed linear and quadratic responses in feed efficiency with increasing Ca levels in the diet of nursery pigs, while Zhou et al. (2023) reported increased ADG in growing–finishing pigs fed 50 μg/kg hydroxyvitamin D3. Although gilts fed the ACaHHyD diet and slaughtered at 100 kg BW had similar initial BW, ADG, and feed efficiency, they unexpectedly had a lower final BW compared with the other groups. Unfortunately, this difference cannot be attributed to dietary effects based on the current experimental design. This is because all gilts received similar nutrients, except for Ca and vitamin D3. As a result, the difference in Ca and vitamin D3 intake is not expected to have a significant impact on the final BW of this group alone.

### Concentrations of Ca and P in plasma and urine

In the diet for gilts weighing between 32 and 100 kg BW, the Ca to digestible P ratios were 3.3 for the high Ca level group and 2.5 for the adequate Ca level group. These ratios did not affect plasma Ca levels, which remained stable between 2.75 and 2.79 mM. However, these levels are slightly greater than the plasma Ca levels (2.32 to 2.55 mM) observed in gilts fed various levels of vitamin D3 and 25-hydroxyvitamin D3 with a fixed Ca level of 8.4 g/kg (Lauridsen et al., 2010). Conversely, in the diet for gilts weighing between 100 and 180 kg BW, the Ca to digestible P ratios were 4.0 for the high Ca level group and 3.0 for the adequate Ca level group. These ratios did not affect the plasma levels of Ca either, although the levels were slightly lower (2.64 to 2.67 mM) than those observed in the diet for 32 to 100 kg gilts. Plasma Ca homeostasis is tightly regulated to maintain Ca within a narrow range (1.74 to 2.72 mmol/L) in both farm mammals and humans (Goldstein, 1990; McDonald et al., 2011). In agreement with this, the plasma Ca content was maintained within a range of 2.64 to 2.79 mmol/L in this study regardless of the dietary treatments and Ca level as well as the age and BW of gilts. An increased serum Ca content was detected in nursery pigs fed the low Ca diet supplemented with 50 μg/kg 25-hydroxyvitamin D3 compared with pigs fed the low Ca diet (Zhang et al., 2022). One of the principal physiological functions of vitamin D3, in combination with hormones, is to maintain homeostasis of Ca and P that supports bone ossification (EFSA, 2009; Goltzman, 2018). The observation that increasing the dietary Ca level has no impact on the digestibility and plasma concentration of Ca and yet substantially increases the urinary concentration of Ca agrees with the hypothesis that Ca homeostasis is mainly regulated at the renal level rather than at the digestive level (Stein et al., 2011).

Plasma concentrations of P were generally greater at 100 kg BW compared to 180 kg BW. The decline in plasma P concentration from 100 to 180 kg BW likely reflects a reduction in the dietary supply of Ca and P. Regardless of the feeding phase (32 to 100 kg or 100 to 180 kg), increased dietary Ca appears to lower plasma P levels. This effect is likely because higher Ca intake can reduce P digestibility (Stein et al., 2011; Lee and Stein, 2022), resulting in decreased plasma P concentrations. Lauridsen et al. (2010) showed that feeding gilts dietary levels of either vitamin D3 or 25-hydroxyvitamin D3, ranging from 200 to 2,000 IU/kg, had no effects on plasma P levels, which is consistent with the present findings.

Regardless of the vitamin D sources, urinary concentration of Ca was greater in urine samples collected at 100 kg BW in gilts supplemented with high Ca levels compared with those fed adequate Ca levels, and the reverse was true for urinary P concentration. Stein et al. (2011) demonstrated a linear increase in urinary excretion of Ca with increasing dietary Ca levels, although the dietary Ca level did not affect the Ca digestibility. Furthermore, these authors revealed a linear decrease in digestibility, absorption, and urinary excretion of P with increasing dietary Ca levels. In line with this, our results also demonstrated a reduced urinary concentration of P at high dietary Ca levels, irrespective of vitamin D sources. Accordingly, it is possible to speculate that a high dietary Ca intake might have negatively impacted P digestibility. This suggestion is supported by the lower plasma P concentration observed in groups supplemented with high dietary Ca, which could indicate reduced P absorption. The differing dietary Ca to digestible P ratios between the adequate and high dietary Ca groups, along with the BW, probably explain the variations in plasma P concentration. In urine samples collected at 180 kg BW, urinary concentration of Ca was greater only in gilts fed the HCaHHyD diet than gilts fed the ACaAD3 diet. On the other hand, urinary concentration of Ca was approximately 1.5- to 3.0-fold greater in urine samples collected at 180 kg BW than at 100 kg BW. This difference may reflect either the difference in the rate of bone resorption, a cumulative effect of Ca level in the diet leading to greater concentration in the urine, or simply just slower growth rate and thereby a need for Ca. It could also be speculated that this outcome resulted from a reduction in ADG and feed efficiency, potentially leading to an increase in Ca intake per kilogram weight gain. In gilts weighing 100 kg and fed adequate Ca diets, the Ca-to-P ratios in urine samples ranged between 0.38 and 0.40 (median). However, these ratios increased in gilts fed high Ca diets. At 180 kg BW, the Ca-to-P ratios became more variable, ranging from 1.99 to 3.92 (median) in gilts fed adequate Ca diets, with a considerable rise in gilts fed high Ca diets compared to their 100 kg counterparts. Substantial variations in Ca-to-P ratios were observed within each treatment group. Some gilts on high Ca diets showed extremely elevated ratios at 100 kg, ranging from 101 to 230. At 180 kg, these high Ca diets resulted in ratios as high as 163 to 259. Due to these considerable variabilities, likely driven by a few gilts with nearly zero urinary P concentrations, we chose not to present the average Ca-to-P ratios even though the use of urinary Ca-to-P ratio was proposed as a plausible indicator of dietary P intake adequacy in sows during gestation and lactation (Grez-Capdeville and Crenshaw, 2021). The cause of these extreme values remains unclear, possibly linked to the time since the last meal or the size of the meal before urine collection. In this study, urinary concentrations of Ca and P, rather than the Ca-to-P ratios, were deemed a more reliable indicator of the gilts’ Ca status. Additionally, the gilts were housed in standard pens, which did not allow for 24 h urine collection. This might have contributed to fewer gilts with extreme Ca-to-P ratios. However, housing them in individual crates would have compromised our aim of assessing bone strength under normal production conditions.

### Vitamin D3 metabolites in plasma

As expected, providing gilts with 50 µg/kg of 25-hydroxyvitamin D3 leads to elevated plasma levels of 25-hydroxyvitamin D3 compared with either 856 or 2,000 IU/kg of vitamin D3. This finding is consistent with other studies (Lauridsen et al., 2010; Lütke-Dörhoff et al., 2023), highlighting that dietary use of 25-hydroxyvitamin D3 is superior to vitamin D3 for maintaining high levels of circulating 25-hydroxyvitamin D3 in plasma. This superiority is due to the greater absorption rates of 25-hydroxyvitamin D3 and the bypassing of liver hydroxylation (Hasan et al., 2023). Moreover, circulating plasma levels of 25-hydroxyvitamin D3 at both 100 and 180 kg BW were considerably greater than those reported by Lauridsen et al. (2010). In contrast, plasma levels of 25-hydroxyvitamin D3 when providing either 856 or 2000 IU/kg of vitamin D3 were comparable to the values in gilts and sows reported by Lauridsen et al. (2010).

With vitamin D3 supplementation, the plasma concentration of 24,25-dihydroxyvitamin D3 at 180 kg relative to 100 kg BW increased by 40% to 58%, compared with only a 16% to 23% increase with 25-dihydroxyvitamin D3 supplementation. The 24,25-dihydroxyvitamin D3 is the degradation product of 25-hydroxyvitamin D3, and it is expected to be excreted via urine (Jones et al., 2012; Zhang and Piao, 2021). Thus, the numerical increase in the concentration of 24,25-dihydroxyvitamin D3 along with the age of the gilts may imply that the degradation rate of this product is affected by the age of the animals and the vitamin D3 sources. A previous study indicated that chondrocytes have a specific uptake of 24,25-dihydroxyvitamin D3 and suggested its involvement in cartilage formation alongside 1,25-dihydroxyvitamin D3 (Corvol et al., 1980). A study in mice indicated that 24,25-dihydroxyvitamin D3 can actively be involved in the repair of bone fracture (St-Arnaud and Naja., 2011). Further investigation is warranted to determine whether the use of 25-hydroxyvitamin D3 may positively affect cartilage since cartilage health affects gait and ultimately, lameness and selection rate.

### Plasma bone turnover biomarkers

In this study, plasma samples were analyzed to explore biomarkers for bone turnover in growing gilts. Bone turnover involves the simultaneous processes of new bone formation by osteoblasts and the resorption of old bone by osteoclasts (Szulc and Bauer, 2013). These processes are intricately balanced to ensure equilibrium between bone removal and formation (Seibel, 2005). Our study focused on assessing the plasma concentrations of osteocalcin, TAP, and BAP as markers for bone formation, and CTX-I as a marker of bone resorption. Irrespective of the dietary treatments and sampling BW, plasma concentrations of osteocalcin and CTX-I remained stable. Although dietary treatment did not show a statistical difference, plasma concentrations of TAP and BAP were numerically greater in plasma samples collected at 100 kg BW than at 180 kg BW. These differences may indicate the age-dependent metabolic activity of the bone rather than dietary responses. In support of this suggestion, Okafor et al. (2024) did not observe a significant effect of either vitamin D3 or 25-hydroxyvitamin D3 supplementations for replacement gilts from 10 wk age until they reached the appropriate age and BW for breeding.

It was hypothesized that exceeding the requirements for Ca and vitamin D3 would affect bone metabolism, leading to potential changes in bone turnover biomarkers, and reduced bone mineralization and strength. Despite dietary treatments affecting the plasma levels of vitamin D3, 25-hydroxyvitamin D3, and P, bone turnover biomarkers did not exhibit compelling evidence to indicate either a beneficial or a negative impact of increased Ca and vitamin D3 supplementations on bone metabolism. However, gilts fed HCaHD3 and slaughtered at 180 kg BW showed a greater plasma concentration of BAP than the remaining groups. Apart from BAP, the remaining bone turnover biomarkers employed in this study do not originate exclusively from bone (Seibel, 2005). Therefore, non-skeletal processes might affect their bloodstream levels, thereby affecting their accuracy as biomarkers. In line with this, in adult humans with normal liver function, approximately 50% of TAP in serum is derived from the liver, with the remaining 50% derived from bone (Green et al., 1971). In a typical bone remodeling cycle, the resorption phase usually spans approximately 10 d, whereas the formation phase extends up to 3 mo (Seibel, 2005). This might suggest a shorter window for capturing revealing biomarkers for bone resorption compared with those for bone formation. Therefore, to comprehensively study the dynamic nature of metabolically active bone tissue, repeated sampling rather than a single sampling might be essential to generate a thorough understanding of bone metabolism.

The literature presents contradictory findings regarding the impact of Ca and/or vitamin D3 supplements on bone metabolism across different production cycles in pigs. For instance, Lauridsen et al. (2010) found no significant impact of dietary vitamin D supplementation on osteocalcin and BAP concentrations in both gilts and sows plasma fed varying levels of vitamin D3 and 25-hydroxyvitamin D3. Similarly, Lütke-Dörhoff et al. (2023) reported no effect of supplementation with either vitamin D3 or 25-hydroxyvitamin D3 (at 50 μg/kg for both) during rearing and fattening on bone metabolism markers concentrations at slaughter. In breeding sows, Tan et al. (2016) reported that concentrations of osteocalcin and pyridinoline remained unaffected when fed varying levels of dietary Ca-to-P ratios from 1 mo after mating until weaning. In growing pigs from 20 kg, a low Ca intake below the requirement (45% and 70%) affected neither plasma nor bone osteocalcin content compared with pigs fed to requirement (Nicodemo et al., 1998). Interestingly, the latter study revealed an inverse relationship between plasma and bone osteocalcin content. It remains unclear whether this inverse relationship is driven by insufficient Ca intake or whether it persists regardless of dietary Ca intake. A linear decrease in the serum content of osteocalcin and BAP with increasing levels of Ca was demonstrated (Carter et al., 1996), while others have elucidated a positive correlation between serum osteocalcin and histomorphometry parameters of bone formation (Lian and Gundberg, 1988; Szulc and Bauer, 2013). The lack of treatment effects on plasma biomarkers in the present study does not preclude the process of bone formation. Analyses of biomarkers and histomorphometry on bone samples could have added to our understanding, but these were not addressed in this study. Evidence linking high serum osteocalcin and BAP contents with increased bone formation mainly originates from studies conducted during the early growth of the animals.

### Bone compositions, measurements, and strength

Regardless of the BW at slaughter, the ratio of Ca to P in bone consistently fell within a narrow range (2.11 to 2.18). This range is comparable to findings in pigs fed varying Ca and P levels and slaughtered at approximately 83 kg (Lagos et al., 2023), as well as in gilts slaughtered 28 d after service (Lauridsen et al., 2010). Lagos et al. (2023) argued that such consistency can be explained by the predominant storage of Ca and P in hydroxyapatite phase, which typically has a Ca-to-P ratio of 2.1:1. Calcium is a key mineral essential for regulating bone mineralization and development. Despite a 27% increase in Ca supply and a 2.3-fold rise in vitamin D3 intake, dietary treatments revealed neither a positive nor an adverse effect on bone Ca and P contents, Ca-to-P ratios, bone dry weight, thickness, or length. However, bone samples from gilts fed a HCaAD3 diet and slaughtered at 100 kg BW had greater ash content than those fed ACaAD3 and ACaHHyD diets. Lee et al. (2021) found greater ash content in the metacarpal bones of pigs when fed a diet that met both Ca and P requirements, in contrast to those fed only 60% of the requirements. A recent study investigated the impact of analytical methods (defatted vs. non-defatted bones) on the assessment of bone mineralization (Williams et al., 2023). This study indicated the importance of employing defatted bone samples when analyzing bone mineralization, typically when reporting bone ash content in absolute terms rather than relative terms. In the present study, we employed non-defatted bone samples, and as a result, the reported values may have been affected to some extent. Gilts fed high Ca and either adequate or high vitamin D3 diets and slaughtered at 100 kg BW showed a tendency toward greater bone-breaking strength than the remaining groups. This difference is likely attributable to the variance in bone ash content among the respective diets. Pearsons’ correlation coefficients indicated a positive correlation between ADG of gilts weighing between 32 and 100 kg BW and bone strength. This correlation is likely due to the fixed day of slaughter, which results in heavier animals having a higher, albeit not significantly higher, bone mass compared with lighter animals. Similar to our findings, supplementation of 50 or 100 μg/kg 25-hydroxyvitamin D3 did not affect bone parameters (dry weight, density, ash, Ca, and P) of the third metacarpal bone (Regassa et al., 2015; Duffy et al., 2018). However, greater bone mineral content and strength were observed in the 3^rd^ and 4^th^ metacarpal bones in pigs supplemented with 50 μg/kg 25-hydroxyvitamin D3 (Zhang et al., 2021; Zhou et al., 2023) compared with those fed 2,000 IU/kg vitamin D3. Increased ash content and bone strength were demonstrated when vitamin D3 was supplemented in doses larger than 800 IU/kg, compared with the same level of 25-hydroxyvitamin D3 (Lauridsen et al., 2010). In the present study, a significantly negative correlation was detected between the plasma concentration of 25-hydroxyvitamin D3 and bone strength in gilts at 180 kg BW that had received dietary concentration of vitamin D3. However, this effect was not significant when the diet contained 25-hydroxyvitamin D3. The reason for this remains unclear, particularly since there was a tendency for lower bone ash content with increasing plasma levels of 25-hydroxyvitamin D3 in gilts fed vitamin D3, whereas gilts fed 25-hydroxyvitamin D3 showed an increase in bone ash content with higher plasma 25-hydroxyvitamin D3 levels. A recent review by Hasan et al. (2023) concluded that supplements containing 25-hydroxyvitamin D3 are more effective than vitamin D3 in improving bone mineralization, although we did not observe such a benefit in the present study. Intake of Ca below the requirement (45% and 70%) led to a considerable reduction in bone mineralization compared with those fed to requirement (Nicodemo et al., 1998).

### Lameness

Previous studies have shown that a high growth rate is associated with increasing lameness (Jørgensen and Sørensen, 1998; Quinn et al., 2015). However, in the present study, no differences were observed in ADG or lameness score of the gilts. Osteochondrosis, a contributing factor to lameness, was not assessed in this study. In the present study, all gilts were fed ad libitum from weaning to 30 kg and then restricted from 30 kg BW onwards, a feeding strategy previously recommended to minimize osteochondrosis in gilts (de Koning et al., 2013). The replacement of dietary vitamin D3 with 25-hydroxyvitamin D3 has been associated with a decreased proportion of young sows showing gait changes and mild lameness, with similar results found in slaughter pigs (Lütke-Dörhoff et al., 2023). However, the current dietary approaches showed no significant effects on lameness or selection rate of the gilts. In line with the current results, Okafor et al. (2024) found no difference in leg structural traits and lameness among replacement gilts fed either 2,000 IU/kg vitamin D3 or 50 μg/kg 25-hydroxyvitamin D3 from 30 kg BW until breeding at 140 kg BW. Okafor et al. (2024) reported selection rates comparable with those in the present study, noting a similar decrease over time. However, in Okafor et al. (2024) gilts that had not been selected were culled at each selection age (at weeks 22, 27, and 35), leaving only the best gilts. This should have led to considerably higher selection rates compared with those in the present study, where all gilts were kept in the study irrespective of the outcome of the initial selection.

The gilt selection rate is influenced by multiple factors, including genetic lines, which can limit the relevance of comparisons between different studies. The issues related to locomotor problems are a notable cause of early culling among breeding sows, presenting both economic and welfare challenges. Modern breeding practices have focused on maximizing litter sizes, placing substantial demands on sows to produce ample amounts of milk to nurse the super-numerous piglets. Consequently, sows often utilize their body reserves during lactation, potentially leading to increased bone resorption and posing difficulties in maintaining optimal leg soundness. Ensuring the provision of essential minerals such as Ca and P becomes crucial in supporting proper bone mineralization, consequently mitigating the risks associated with leg weakness. A slight decrease in leg swelling prevalence was observed in sows supplemented with 50 μg/kg 25-hydroxyvitamin D3 compared with those receiving 2,000 IU/kg standard vitamin D3 (Lütke-Dörhoff et al., 2023), although we did not see a similar effect in the present study. Despite the absence of notable effects of dietary treatments on lameness, it is important to note that the lameness score tended to be higher in gilts slaughtered at 180 kg BW than those slaughtered at 100 kg BW. Furthermore, the Pearsons’ correlation coefficient for gilts weighing between 100 and 180 kg BW revealed a negative correlation between ADG and lameness score (*r* = -0.25). This indicates that lameness decreased as ADG increased. However, no such effect was observed in gilts weighing between 32 and 100 kg BW.

## Conclusion

The present study demonstrated that the growth performance of the gilts and their plasma Ca concentration remained unaffected by varying levels of dietary Ca and levels and sources of vitamin D3 from adequate to high. This study implied that high levels of dietary Ca negatively impacted P utilization as lower plasma concentrations were observed in gilts supplemented with high dietary Ca level. It was evident that plasma concentrations of vitamin D3 and its main metabolites were significantly increased by dietary supplementation of vitamin D3 although this did not improve bone quality parameters, lameness score, or selection rate of the gilts. Overall, higher Ca and vitamin D supplementation slightly increased bone ash content but had no effect on lameness or selection rate of the gilts compared to those fed according to the Danish nutrient standards.

## Supplementary Material

skae310_suppl_Supplementary_Table_S1

## Abbreviations

ADG: average daily gain
BW: body weight
BAP: bone-specific alkaline phosphatase
Ca: calcium
CTX-I: C-terminal cross-linking telopeptide of type I Collagen
ME: metabolizable energy
P: phosphorus
TAP: total alkaline phosphatase

## References

[CIT0001] Ala-Kurikka, E., M. Heinonen, K. Mustonen, O. Peltoniemi, M. Raekallio, O. Vainio, and A. Valros. 2017. Behavior changes associated with lameness in sows. Appl. Anim. Behav. Sci. 193:15–20. doi: https://doi.org/10.1016/j.applanim.2017.03.017

[CIT0002] Anil, S. S., L. Anil, and J. Deen. 2009. Effect of lameness on sow longevity. J. Am. Vet. Med. Assoc. 235:734–738. doi: https://doi.org/10.2460/javma.235.6.734 19751172

[CIT0003] Bonde, M., T. Rousing, J. H. Badsberg, and J. T. Sørensen. 2004. Associations between lying-down behaviour problems and body condition, limb disorders and skin lesions of lactating sows housed in farrowing crates in commercial sow herds. Livest. Prod. Sci. 87:179–187. doi: https://doi.org/10.1016/j.livprodsci.2003.08.005

[CIT0004] Carter, S. D., G. L. Cromwell, T. R. Combs, G. Colombo, and P. Fanti. 1996. The determination of serum concentrations of osteocalcin in growing pigs and its relationship to end-measures of bone mineralization. J. Anim. Sci. 74:2719–2729. doi: https://doi.org/10.2527/1996.74112719x 8923186

[CIT0005] Corvol, M., A. Ulmann, and M. Garabedian. 1980. Specific nuclear uptake of 24,25-dihydroxycholecalciferol, a vitamin D3 metabolite biologically’ active in cartilage. FEBS Lett. 116:273–276. doi: https://doi.org/10.1016/0014-5793(80)80661-2 6967830

[CIT0006] Danfær, A. and A. B. Strathe. 2012. Quantitative and physiological aspects of pigs growth. In: Bach Knudsen, N. J. K. K. E., H. D. Poulsen, and B. B. Jensen, editors. Text book of physiology [in Danish: Læreborg I fysiology]. Denmark: SEGES Innovation.

[CIT0056] de Koning, D. B., E. M. van Grevenhof, B. F. A. Laurenssen, P. R. van Weeren, W. Hazeleger, and B. Kemp. 2013. The influence of dietary restriction before and after 10 weeks of age on osteochondrosis in growing gilts1. J. Anim. Sci. 91:5167–5176. doi: https://doi.org/10.2527/jas.2013-6591 23989871

[CIT0007] Denu, L., R. Goessl, and P. Hofmann. 2008. Method for the determination of 25-Hydroxy cholecalcferol in feed. United States patent US 7,358,092 B2. April 15, 2008.

[CIT0008] Duffy, S. K., A. K. Kelly, G. Rajauria, L. C. Clarke, V. Gath, F. J. Monahan, and J. V. O’Doherty. 2018. The effect of 25-hydroxyvitamin D(3) and phytase inclusion on pig performance, bone parameters and pork quality in finisher pigs. J. Anim. Physiol. Anim. Nutr. 102:1296–1305. doi: https://doi.org/10.1111/jpn.12939 29974992

[CIT0009] EFSA. 2009. Scientific Opinion on the substantiation of health claims related to vitamin D and maintenance of bone and teeth (ID 150, 151, 158), absorption and utilisation of calcium and phosphorus and maintenance of normal blood calcium concentrations (ID 152, 157), cell division (ID 153), and thyroid function (ID 156) pursuant to Article 13(1) of Regulation (EC) No 1924/20061. EFSA J. 7:19. doi: https://doi.org/10.2903/j.efsa.2009.1227

[CIT0010] European-Commission. 2009. Commission Regulation (EC) No 152/2009 of 27 January 2009 laying down the methods of sampling and analysis for the official control of feed. Off. J. Eur. Union L. 54:1.

[CIT0058] EvaPig. 2020. Equations and coefficients. [accessed July 15, 2021]. https://en.evapig.com/resources/media/EvaPig_ManualEquations.pdf.

[CIT0012] Goldstein, D. A. 1990. Serum calcium. In: H. K. Walker, W. D. Hall and J. W. Hurst, editors, Clinical methods: the history, physical, and laboratory examination. Boston, USA: Butterworth Publishers. 21250045

[CIT0013] Goltzman, D. 2018. Functions of vitamin D in bone. Histochem. Cell Biol. 149:305–312. doi: https://doi.org/10.1007/s00418-018-1648-y 29435763

[CIT0014] Green, S., C. L. Anstiss, and W. H. Fishman. 1971. Automated differential isoenzyme analysis. II. The fractionation of serum alkaline phosphatases into “liver”, “intestinal” and “other” components. Enzymologia. 41:9–26. 5116110

[CIT0015] Grez-Capdeville, M., and T. D. Crenshaw. 2021. Evaluation of calcium to phosphorus ratio in spot urine samples as a practical method to monitor phosphorus intake adequacy in sows. J. Anim. Sci. 99:1. doi: https://doi.org/10.1093/jas/skab335 PMC8648296 34791271

[CIT0016] Hasan, M., M. Oster, H. Reyer, K. Wimmers, and D. -C. Fischer. 2023. Efficacy of dietary vitamin D3 and 25(OH)D3 on reproductive capacities, growth performance, immunity and bone development in pigs. Br. J. Nutr. 130:1298–1307. doi: https://doi.org/10.1017/s0007114523000442 36847163 PMC10511684

[CIT0017] Hu, Y., J. van Baal, J. W. Resin, and P. Bikker. 2022. Dietary calcium supplementation reduces phosphorous absorption and causes a shift from transcellular to paracellular calcium absorption in pigs. Anim. Sci. Proced. 13:188. doi: https://doi.org/10.1016/j.anscip.2022.03.274

[CIT0018] Jones, G., D. E. Prosser, and M. Kaufmann. 2012. 25-Hydroxyvitamin D-24-hydroxylase (CYP24A1): its important role in the degradation of vitamin D. Arch. Biochem. Biophys. 523:9–18. doi: https://doi.org/10.1016/j.abb.2011.11.003 22100522

[CIT0019] Jørgensen, B., and M. T. Sørensen. 1998. Different rearing intensities of gilts: II. Effects on subsequent leg weakness and longevity. Livest. Prod. Sci. 54:167–171. doi: https://doi.org/10.1016/s0301-6226(97)00177-2

[CIT0020] Lagos, L. V., J. C. Woodworth, S. W. Kim, and H. H. Stein. 2023. Short communication: commercial diets for pigs in the United States contain more calcium than formulated. J. Anim. Sci. 101:1–5. doi: https://doi.org/10.1093/jas/skad102 PMC10500971 37707374

[CIT0021] Lauridsen, C., U. Halekoh, T. Larsen, and S. K. Jensen. 2010. Reproductive performance and bone status markers of gilts and lactating sows supplemented with two different forms of vitamin D. J. Anim. Sci. 88:202–213. doi: https://doi.org/10.2527/jas.2009-1976 19783698

[CIT0022] Lee, S. A., and H. H. stein. 2022. Apparent ileal digestibility of amino acids by pigs affected by increasing dietary calcium from deficient to excess concentrations, but phosphorus digestibility is reduced. Anim. Feed Sci. Technol. 292:115436. doi: https://doi.org/10.1016/j.anifeedsci.2022.115436

[CIT0023] Lee, S. A., L. V. Lagos, M. R. Bedford, and H. H. Stein. 2021. Quantities of ash, Ca, and P in metacarpals, metatarsals, and tibia are better correlated with total body bone ash in growing pigs than ash, Ca, and P in other bones. J. Anim. Sci. 99:2139–2144. doi: https://doi.org/10.1093/jas/skab149 PMC8287912 33959745

[CIT0024] Letourneau-Montminy, M. P., A. Narcy, M. Magnin, D. Sauvant, J. F. Bernier, C. Pomar, and C. Jondreville. 2010. Effect of reduced dietary calcium concentration and phytase supplementation on calcium and phosphorus utilization in weanling pigs with modified mineral status. J. Anim. Sci. 88:1706–1717. doi: https://doi.org/10.2527/jas.2008-1615 20118415

[CIT0025] Lian, J. B., and C. M. Gundberg. 1988. Osteocalcin. Biochemical considerations and clinical applications. Clin. Orthop. Relat. Res. 226:267???291–267???291. doi: https://doi.org/10.1097/00003086-198801000-00036 3275514

[CIT0026] Luo, J., H. G. Lei, L. Y. Shen, R. L. Yang, Q. Pu, K. P. Zhu, M. Z. Li, G. Q. Tang, X. W. Li, S. H. Zhang, et al 2015. Estimation of growth curves and suitable slaughter weight of the liangshan pig. Asian-Australas. J. Anim. Sci. 28:1252–1258. doi: https://doi.org/10.5713/ajas.15.0010 26194218 PMC4554864

[CIT0027] Lütke-Dörhoff, M., J. Schulz, H. Westendarp, C. Visscher, and M. R. Wilkens. 2023. Effects of maternal and offspring treatment with two dietary sources of vitamin D on the mineral homeostasis, bone metabolism and locomotion of offspring fed protein- and phosphorus-reduced diets. Arch. Anim. Nutr. 77:42–57. doi: https://doi.org/10.1080/1745039X.2023.2172310 36757473

[CIT0028] McDonald, O., J. F. D. Greenhalgh, C. A. Morgan, L. A. Sinclair, and R. G. Wilkinson. 2011. Animal nutrition. 7th ed. England: Pearson Education Limited.

[CIT0055] Merriman, L. A., C. L. Walk, M. R. Murphy, C. M. Parsons, and H. H. Stein. 2017. Inclusion of excess dietary calcium in diets for 100- to 130-kg growing pigs reduces feed intake and daily gain if dietary phosphorus is at or below the requirement1. J. Anim. Sci. 95:5439–5446. doi: https://doi.org/10.2527/jas2017.1995 29293757 PMC6292265

[CIT0030] Nicodemo, M. L. F., D. Scott, W. Buchan, A. Duncan, and S. P. Robins. 1998. Effects of variations in dietary calcium and phosphorus supply on plasma and bone osteocalcin concentrations and bone mineralization in growing pigs. Exp. Physiol. 83:659–665. doi: https://doi.org/10.1113/expphysiol.1998.sp004147 9793786

[CIT0031] O’Doherty, J. V., D. A. Gahan, C. O’Shea, J. J. Callan, and K. M. Pierce. 2010. Effects of phytase and 25-hydroxyvitamin D3 inclusions on the performance, mineral balance and bone parameters of grower-finisher pigs fed low-phosphorus diets. Animal. 4:1634–1640. doi: https://doi.org/10.1017/s1751731110000807 22445115

[CIT0032] Okafor, P. C. J., N. Jimongkolkul, A. Khongparadit, W. Ahiwichai, and N. Homwong. 2024. Enhancement of selectivity, 25-hydroxyvitamin D3 level, alkaline phosphatse activity and reprodctive performance in gilts and primiparous sows using 14-epimer of 25-hydroxyvitamin D3. Vet. Anim. Sci. 24:1–13. doi: https://doi.org/10.1016/j.vas.2024.100352 PMC11064612 38699218

[CIT0033] Patience, J. F. 2012. The influence of dietary energy on feed efficiency in grow-finish swine. In: J. F. Patience, editor, Feed efficiency in swine. The Netherlands: Wageningen Academic Publishers; p. 101–129.

[CIT0034] Pluym, L. M., A. Van Nuffel, S. Van Weyenberg, and D. Maes. 2013. Prevalence of lameness and claw lesions during different stages in thereproductive cycle of sows and the impact on reproduction results. Animal. 7:1174–1181. doi: https://doi.org/10.1017/S1751731113000232 23714359 PMC3666190

[CIT0035] Quinn, A. J., L. E. Green, P. G. Lawlor, and L. A. Boyle. 2015. The effect of feeding a diet formulated for developing gilts between 70 kg and ~140 kg on lameness indicators and carcass traits. Lives. Sci. 154:87–95. doi: https://doi.org/10.1016/j.livsci.2014.12.016

[CIT0036] Regassa, A., R. Adhikari, C. M. Nyachoti, and W. K. Kim. 2015. Effects of 25-(OH)D3 on fecal Ca and P excretion, bone mineralization, Ca and P transporter mRNA expression and performance in growing female pigs. J. Environ. Sci. Health B. 50:293–299. doi: https://doi.org/10.1080/03601234.2015.999612 25714461

[CIT0037] Rider, S., V. Verlhac-Trichet, D. Constant, E. Chenal, S. Etheve, B. Riond, H. Schmidt-Posthaus, and R. Schoop. 2023. Calcifediol is a safe and effective metabolite for raising vitamin D status and improving growth and feed conversion in rainbow trout. Aquaculture. 568:739285. doi: https://doi.org/10.1016/j.aquaculture.2023.739285

[CIT0038] Schadt, H. S., R. Gössl, N. Seibel, and C. -P. Aebischer. 2019. Quantification of vitamin D3 in feed, food, and pharmaceuticals using high-performance liquid chromatography/tandem mass spectrometry. J. AOAC Int. 95:1487–1494. doi: https://doi.org/10.5740/jaoacint.11-512 23175984

[CIT0039] Schlegel, P., and A. Gutzwiller. 2020. Dietary calcium to digestible phosphorus ratio for optimal growth performance and bone mineralization in growing and finishing pigs. Animals. 10:178. doi: https://doi.org/10.3390/ani10020178 31973009 PMC7070681

[CIT0040] Seibel, M. J. 2005. Biochemical markers of bone turnover: part I: biochemistry and variability. Clin. Biochem. Rev. 26:97–122. 16648882 PMC1320175

[CIT0041] St-Arnaud, R., and R. P. Naja. 2011. Vitamin D metabolism, cartilage and bone fracture repair. Mol. Cell. Endocrinol. 347:48–54. doi: https://doi.org/10.1016/j.mce.2011.05.018 21664253

[CIT0042] Stein, H. H., O. Adeola, G. L. Cromwell, S. W. Kim, D. C. Mahan, and P. S. Miller; North Central Coordinating Committee on Swine Nutrition (NCCC-42). 2011. Concentration of dietary calcium supplied by calcium carbonate does not affect the apparent total tract digestibility of calcium, but decreases digestibility of phosphorus by growing pigs. J. Anim. Sci. 89:2139–2144. doi: https://doi.org/10.2527/jas.2010-3522 21335534

[CIT0043] Szulc, P., and D. C. Bauer. 2013. Biochemical markers of bone turnover in osteoporosis. In: R. Marcus, D. Feldman, D. Dempster, M. Luckey and J. A. Cauley, editors, Amsterdam: Osteoporosis ELSEVIER; p. 1573–1610.

[CIT0044] Tan, F. P. Y., S. A. Kontulainen, and A. D. Beaulieu. 2016. Effects of dietary calcium and phosphorus on reproductive performance and markers of bone turnover in stall- or group-housed sows. J. Anim. Sci. 94:4205–4216. doi: https://doi.org/10.2527/jas.2016-0298 27898869

[CIT0045] Tybirk, p., N. M. Sloth, and K. Blaabjerg. 2021. Danish nutrient standards. Denmark: SEGES Danish Pig Research Centre. [accessed 14 October 2023] https://pigresearchcentre.dk/Nutrient-standards

[CIT0046] Tybirk, P., T. S. Bruun, and G. Sørensen. 2014. New nutrient standards for gilts and sows in the breeding section]. [In Danish: Ny næringsstofnopmer til polte og søer in løbeafdeling]. Publication no. 1413, Copenhagen, Denmark: SEGES Innovation. Nye næringsstofnormer til polte og søer i løbeafdeling (svineproduktion.dk) [accessed 21 June 2024

[CIT0047] van Riet, M. M. J., S. Millet, M. Aluwé, and G. P. J. Janssens. 2013. Impact of nutrition on lameness and claw health in sows. Livest. Sci. 156:24–35. doi: https://doi.org/10.1016/j.livsci.2013.06.005

[CIT0048] Vestergaard, K., M. G. Christiansen, and L. U. Hansen. 2016. Analysis of sows mortality in 17 Danish herd [In Danish “Analyse af sodødelighed i 17 danske besætninger”]. Copenhage, Denmark: Videncenter for Svineproduktion, Notat nr: 1604. Available at: https://svineproduktion.dk/-/media/PDF---Publikationer/Notater-2016/Notat_1604.ashx

[CIT0049] Willgert, K. J. E., V. Brewster, A. J. Wright, and A. Nevel. 2014. Risk factors of lameness in sows in England. Prev. Vet. Med. 113:268–272. doi: https://doi.org/10.1016/j.prevetmed.2013.10.004 24331733

[CIT0050] Williams, H. R., T. E. Chin, M. D. Tokach, J. C. Woodworth, J. M. DeRouchey, R. D. Goodband, J. R. Bergstrom, M. C. Rahe, C. L. Siepker, P. Sitthicharoenchai, et al 2023. The effect of bone and analytical methods on the assessment of bone mineralization response to dietary phosphorus, phytase, and vitamin D in nursery pigs. J. Anim. Sci. 101:1–15. doi: https://doi.org/10.1093/jas/skad353 PMC10635674 37837391

[CIT0051] Zhai, H. X., J. Bergstrom, J. C. Zhang, W. Dong, Z. Z. Wang, K. Stamatopoulos, and A. J. Cowieson. 2023. The effects of increasing dietary total Ca/total P ratios on growth performance, Ca and P balance, and bone mineralization in nursery pigs fed diets supplemented with phytase. Transl. Anim. Sci. 7:1–8. doi: https://doi.org/10.1093/tas/txad006 PMC9977215 36873609

[CIT0052] Zhang, L., M. Yang, and X. Piao. 2022. Effects of 25-hydroxyvitamin D(3) on growth performance, serum parameters, fecal microbiota, and metabolites in weaned piglets fed diets with low calcium and phosphorus. J. Sci. Food Agric. 102:597–606. doi: https://doi.org/10.1002/jsfa.11388 34148242

[CIT0053] Zhang, L. H., H. S. Liu, S. J. Liu, and X. S. Piao. 2021. Dietary supplementation with 25-hydroxycholecalciferol and phytase in growing-finishing pigs: II. Effects on intestinal antioxidant status, immunity and bone quality. Anim. Feed Sci. Technol. 280:115065–115010. doi: https://doi.org/10.1016/j.anifeedsci.2021.115065

[CIT0054] Zhou, X., L. Wang, Z. Zhang, X. Qin, B. Qiu, J. Cao, D. Han, J. Wang, and J. Zhao. 2023. 25-Hydroxyvitamin D3 improved growth performance, bone characteristics and polyunsaturated fatty acid deposition by activating calcium ion channel proteins expression in growing pigs. J. Funct. Foods. 105:105581–105589. doi: https://doi.org/10.1016/j.jff.2023.105581

