# Supplementary material for: Effect of dietary calcium and vitamin D supplements on plasma bone turnover biomarkers, bone mineralization, bone strength, and lameness score in gilts
Source: J Anim Sci. 2024 Oct 15;102:skae310. doi: 10.1093/jas/skae310 (PMC11568344; doi:10.1093/jas/skae310)
Supplement: skae310_suppl_Supplementary_Table_S1 [file skae310_suppl_supplementary_table_s1.docx]

**Table 1 Supplement.** The daily feeding schemes used in the present study regardless of the dietary treatments. The feed allowance increased gradually every week, but dietary compositions were changed at the time of the first slaughter when gilts reached 100 kg body weight, indicating the decline in dietary lysine requirement of the gilts after 100 kg body weight.

| Age, days | Expected body weight, kg | Feed level, kg/day | Dietary lysine level, g SID Lys/day |
| --- | --- | --- | --- |
| 77 | 30.0 | 1.40 | 9.44 |
| 84 | 33.9 | 1.43 | 9.64 |
| 91 | 38.3 | 1.54 | 10.38 |
| 98 | 43.0 | 1.68 | 11.32 |
| 105 | 48.2 | 1.82 | 12.27 |
| 112 | 53.7 | 1.96 | 13.21 |
| 119 | 59.7 | 2.10 | 14.15 |
| 126 | 66.0 | 2.24 | 15.10 |
| 133 | 72.5 | 2.38 | 16.04 |
| 140 | 79.2 | 2.52 | 16.98 |
| 147 | 86.1 | 2.62 | 17.66 |
| 154 | 92.9 | 2.66 | 17.93 |
| 161 | 99.6 | 2.71 | 18.27 |
| 168 | 106.1 | 2.71 | 18.27 |
| 175 | 112.4 | 2.71 | 11.79 |
| 182 | 117.7 | 2.71 | 11.79 |
| 189 | 122.9 | 2.71 | 11.79 |
| 273 | 183.8 | 2.71 | 11.79 |
